# Differentiating mpox infection and vaccination using a validated multiplex orthopoxvirus IgG serology assay

**DOI:** 10.1128/jcm.01548-25

**Published:** 2025-12-29

**Authors:** Jonathan C. Reed, Cecilia Downs, Kaden McAllister, Clarice Mauer, Christopher L. McClurkan, Donna Wilson, Kate Holzhauer, Jane A. Dickerson, Chase A. Cannon, Tara M. Babu, Matthew R. Golden, David M. Koelle, Alexander L. Greninger

**Affiliations:** 1 Department of Laboratory Medicine and Pathology, University of Washington Medical Center 7284, Seattle, Washington, USA; 2 Department of Laboratories, Seattle Children's Hospital 7274, Seattle, Washington, USA; 3 Division of Allergy and Infectious Diseases, Department of Medicine, University of Washington 7284, Seattle, Washington, USA; 4 Public Health-Seattle & King County, HIV/STI/HCV Program 7285, Seattle, Washington, USA; 5 Department of Epidemiology, University of Washington 7284, Seattle, Washington, USA; 6 Vaccine and Infectious Disease Division, Fred Hutchinson Cancer Research Center 7286 , Seattle, Washington, USA; 7 Department of Global Health, University of Washington 7284, Seattle, Washington, USA; 8 Benaroya Research Institute 128776, Seattle, Washington, USA; Mayo Clinic Minnesota, Rochester, Minnesota, USA

**Keywords:** multi-antigen panel, mpox, serology, vaccinia, MPXV, monkeypox, orthopox, orthopoxvirus

## Abstract

The resurgence of monkeypox virus (MPXV) has increased demand for validated serological assays to assess exposure and immunity. Cross-reactivity among orthopoxviruses, stemming from high sequence conservation, complicates distinguishing antibody responses from natural MPXV infection versus vaccination or other orthopoxvirus exposures. We validated the Meso Scale Discovery (MSD) V-PLEX Orthopoxvirus Panel 1 (IgG) Kit, which quantifies antibody levels to five MPXV antigens and their vaccinia virus (VACV) orthologs, following Good Clinical Laboratory Practice guidelines. We assessed assay performance using serum from 26 individuals with prior mpox, 52 JYNNEOS vaccine recipients, and 179 unexposed controls. The assay reliably detected antibody responses in all exposed cohorts with peak levels observed 2 months post-vaccination. Antibody levels to specific antigens also correlated with Modified Vaccinia Ankara neutralization titer, particularly for MPXV B6R/VACV B5R, MPXV E8L/VACV D8L, and MPXV M1R/VACV L1. Receiver operating characteristic analysis showed that some individual antigens achieved high sensitivity and specificity for exposure detection (area under the curve [AUC] > 0.96 for VACV D8L, MPXV B6R, VACV B5R); however, individual antigens performed poorly in distinguishing infection from vaccination. In contrast, antibody level ratios between some MPXV and VACV orthologs effectively differentiated MPXV infection from vaccinia vaccination with high sensitivity and specificity (e.g., MPXV A35R/VACV A33R ortholog ratio, AUC = 0.97, sensitivity = 0.97, specificity = 0.96). Our findings validate the MSD assay for clinical research and serosurveillance to assess MPXV immunity and support the utility of ortholog pair ratio analysis as a strategy to discriminate vaccinated and infected individuals.

**IMPORTANCE:**

Mpox continues to spread around the world, with recent data showing increasing incidence in the United States. While there are multiple Food and Drug Administration (FDA)-authorized real-time PCR tests for diagnostic use, there are no FDA-authorized serological tests and few laboratory-developed serological tests offered. We evaluated the Meso Scale Discovery V-PLEX Orthopoxvirus Panel 1 (IgG) Kit according to Good Clinical Laboratory Practice guidelines and found that the assay reliably detected antibody responses in monkeypox virus (MPXV)- and vaccinia virus (VACV)-exposed cohorts and could distinguish them from unexposed cohorts. Intriguingly, we found that antibody level ratios between certain MPXV and VACV orthologs could distinguish prior mpox infection from vaccinia vaccination. Overall, these data highlight the use of multi-antigen panels in challenging scenarios for serological testing, such as the cross-reactivity presented by orthopoxviruses.

## INTRODUCTION

Monkeypox virus (MPXV) is an enveloped, double-stranded DNA virus that belongs to the *Orthopoxvirus* genus of the *Poxviridae* family and is the causative agent of mpox (1). In 2022, the Food and Drug Administration (FDA) and European Medicines Agency (EMA) approved the use of tecovirimat for the treatment of mpox, though the drug has since failed to show clinical efficacy in three Phase III randomized control trials (2, 3). Vaccines based on live-attenuated vaccinia virus (VACV) induce strong cross-protective immunity against orthopoxviruses and were central to the global eradication of *Orthopoxvirus variola virus* (smallpox). Modern third-generation modified live-attenuated VACV vaccines, marketed as JYNNEOS or IMVANEX, are approved by both the FDA and EMA for the prevention of mpox and as post-exposure prophylaxis in adults aged 18 years and older (4, 5).

As of 31 August 2025, the global outbreak of mpox has resulted in more than 150,000 cases and 424 deaths (6). Diagnosis typically relies on PCR-based assays (7), though serological testing could aid in identifying asymptomatic infections, estimating population immunity, defining correlates of protection, and discriminating people with prior mpox from VACV-vaccinated individuals. Currently, no FDA-authorized serology test exists for mpox diagnosis or immunity, and only three mpox serology tests are currently being offered clinically in the United States—ELISAs for orthopoxvirus IgG or IgM and a microsphere-based immunoassay for orthopoxvirus IgG antibody detection (8, 9). High sequence similarity (94%–98%) between MPXV and VACV surface proteins complicates serological discrimination due to cross-reactivity (10). In 2008, adsorption-based ELISA or Western blot was shown to distinguish between MPXV-infected versus VACV-vaccinated individuals, albeit in small populations (11, 12). Single-analyte ELISA or whole-virion serological tests have not been able to adequately separate these populations, though efforts to identify discriminatory antigens are ongoing (13–15).

Multi-antigen serological tests may have a greater ability to discriminate among these populations. ELISA testing, separately conducted with 24 MPXV and 3 VACV antigens, suggested MPXV A27L as a marker of MPXV infection and MPXV M1R (VACV L1R) as an indicator of JYNNEOS vaccination (15). A multiplexed microsphere immunoassay consisting of two MPXV-specific peptides and five cross-reactive OPV antigens demonstrated >90% sensitivity and specificity in detecting people with prior mpox in vaccinated populations (16). Likewise, in a Luminex-based assay incorporating nine MPXV antigens and three VACV antigens, a panel of eight of these antigens collectively distinguished people with prior mpox or VACV-vaccinated individuals from unexposed controls with 98% sensitivity and 95% specificity. Among these positives, MPXV A27L was then used to distinguish MPXV-infected from VACV-vaccinated with a sensitivity of 88% and a specificity of 97% (14). However, neither of these assays is commercially available for clinical labs to run in-house.

The main commercially available multi-antigen panel for MPXV binding serology is the Meso Scale Discovery (MSD) V-PLEX Orthopoxvirus Panel 1 (IgG) Kit. Prior evaluations of the kit have shown that MPXV M1/VACV L1 antibody levels were higher on average in IMVANEX-vaccinated individuals compared to unexposed controls, consistent with previous findings (15, 17). Furthermore, MPXV M1/VACV L1 demonstrated greater sensitivity and specificity in distinguishing sera from vaccinated individuals from unexposed controls than in differentiating sera from people with prior mpox from unexposed controls. A similar trend was observed for MPXV A29L/VACV A27L, but in the opposite direction, showing improved performance in identifying people with prior mpox relative to unexposed controls (17). Among the two MSD studies, MPXV E8L performed best (or tied) to discriminate exposed (vaccinated or MPXV-infected) from unexposed (13, 17). Additionally, MSD antibody level results have been compared to MPXV- or VACV-neutralizing activity, but were not found to be strongly correlated (18). Here, we established the performance characteristics of the MSD V-PLEX Orthopoxvirus Panel 1 (IgG) Kit, including comparison to a Modified Vaccinia Ankara (MVA)-neutralizing antibody test, using sera taken from people with prior mpox, JYNNEOS vaccine recipients, a pediatric control cohort, and otherwise healthy adults under 40 years of age screened for anti-rubella virus IgG antibodies.

## MATERIALS AND METHODS

### Specimens and cohorts

Several cohorts of specimens were acquired for this study, and their demographic information is summarized in Table 1.

**TABLE 1:** Summary of demographic characteristics for each cohort^*a*^

	Pediatric (*n* = 68)	Rubella IgG^+^ (*n* = 111)	Vaccine cohort A (*n* = 22)	Vaccine cohort B (*n* = 30)	MPXV 2-6 mo (*n* = 9)	MPXV 10 mo (*n* = 17^*c*^)
Male, n (%)	35 (51%)	14 (13%)	17 (77%)	30 (100%)	9 (100%)	17 (100%)
Female, n (%)	33 (49%)	97 (87%)	5 (23%)	0 (0%)	0 (0%)	0 (0%)
Age, mean (range)	9 (4–15)	32 (19–42)	37 (20–75)	36 (20–65)	36 (31–42)	32 (20–49)
HIV positive, n (%)	NA^*d*^	NA	1 (5%)	0 (0%)	7 (78%)	0 (0%)
Born < 1972, n(%)	0 (0%)	0 (0%)	4 (18%)	2 (7%)	0 (0%)	0 (0%)
History of smallpox vaccination, n (%)	0 (0%)	0 (0%)	6 (27%)	0 (0%)	0 (0%)	0 (0%)
Months post-MPXV, mean (range)	NA	NA	NA	NA	4.2 (1.9–5.6)	10.3 (7.9–12.4)
Months post-vaccine, mean (range)	NA	NA	Multiple collections^*b*^	9.1 (5.5–10.9)	NA	NA

^*a*^ months (mo).

^*b*^ Specimens were collected at baseline, 1 mo after first dose, and 2 mo after first dose.

^*c*^ One individual was sampled twice, once at 9.6 months and once at 11.6 months post-MPXV infection, so total number of specimens analyzed was 18.

^*d*^ NA, not applicable.

Two cohorts presumed to be unexposed to orthopoxviruses and immunocompetent were included as negative controls for assay specificity and threshold determination. The first cohort consisted of 111 deidentified remnant serum samples from adults born after 1981 with detectable anti-rubella IgG (Rubella IgG+ cohort) on the FDA-approved DiaSorin LIAISON Rubella IgG Assay, obtained from the University of Washington (UW) Virology Laboratory. Sera positive for anti-rubella IgG were selected to represent a generally healthy population with evidence of prior immune response and immunocompetence. The second cohort included 68 deidentified remnant serum samples from healthy pediatric patients undergoing allergen testing at Seattle Children’s Hospital (Pediatric cohort). Individuals in both cohorts are not expected to have received the live-attenuated VACV vaccine for smallpox, as routine smallpox vaccination ended in the United States by 1972. This was important because prior VACV vaccination induces antibody responses detectable by the assay. Furthermore, neither cohort was expected to have experienced natural orthopoxvirus infection, particularly the pediatric group.

Two cohorts were acquired to evaluate responses associated with JYNNEOS smallpox vaccination. The longitudinal vaccine cohort (Vaccine A cohort) comprised 22 individuals with a complete set of serum specimens collected at baseline (Vaccine A-0 month), 1 month after the first dose (Vaccine A-1 month), and 1 month after the second dose (Vaccine A-2 months), provided by the UW Virology Research Clinic (VRC). The cross-sectional vaccine cohort (Vaccine B) included 30 individuals sampled at an average of 9.1 months post-vaccination, provided by the Sexual Health Clinic (SHC) at Public Health-Seattle & King County (PHSKC).

Lastly, MPXV responses were assessed in two cohorts. The first cohort (MPXV-2–6 months) consisted of nine individuals sampled at an average of 4.2 months post-infection, provided by the UW VRC. The second cohort (MPXV-10 months) included 17 individuals sampled at an average of 10.3 months post-infection, provided by the SHC at PHSKC(19). One individual in the MPXV-10-month cohort was sampled twice (at 9.6 and 11.6 months), resulting in a total of 18 specimens.

Medical chart review of individuals in the Vaccine A and MPXV-2- to 6-month cohorts revealed no evidence of immunosuppression. Furthermore, individuals living with HIV-1 in these cohorts had CD4 counts >300 cells/mm³, indicating immunocompetence. For the MPXV 10-month and Vaccine B cohorts, none of the participants were known to be HIV-1 positive and are presumed immunocompetent.

Specimen use was approved by the University of Washington or Seattle Children’s Hospital Institutional Review Board with a consent waiver for control cohorts of remnant specimens and with written informed consent from the VRC and SHC/PHSKC cohorts.

### VACV focus reduction neutralization test

Flat-bottomed 96-well plates (Corning, 3585) were seeded with 10,000 VeroE6 cells (ATCC, CRL-1586) per well in 100 µL DMEM-10, which consisted of DMEM (Gibco, 10564-011), supplemented with 10% fetal bovine serum (Gibco, A38400-01) and 1% Penicillin–Streptomycin (Gibco, 15140-122) and incubated overnight at 37°C in 5% CO_2_.

Serum specimens were thawed and heat-inactivated at 56°C for 30 min. 10.5 µL of sera was diluted into 94.5 µL of DMEM-10 in a polypropylene U-bottomed plate (Thermo Fisher, 267245) with one column left with only DMEM-10 to serve as a virus-only control (VOC). A row at the top and bottom of the plates serves as media-only controls (MO). Sera were then serially threefold diluted by transferring 35 µL 1:10 diluted sera into 70 µL complete DMEM-10, creating dilutions ranging from 10-fold to 2,430-fold. A 50 µL aliquot of the expanded MVA P1 passage (Supplementary Material) was sonicated on ice using a cup horn at 50% power with 2-s pulse on/2 s pulse off for 1 min and then diluted 1,500-fold in complete DMEM-10. Then 70 µL of diluted virus was added to 70 µL diluted sera and VOC wells, diluting sera a further 2-fold, for a final range of 20-fold to 4,860-fold. The virus/serum mixture was incubated at 37°C for 1 h, the media was aspirated from pre-plated VeroE6 cells, and 50 µL of the virus/serum mixes, VOC controls, and MO controls were added to VeroE6 cells in duplicate and incubated for 24 h at 37°C. Some specimens were initially diluted by combining 21 µL of sera and 84 µL of complete DMEM-10 (final dilution series 10-fold to 2,430-fold) or by combining 5.25 µL of sera and 99.75 µL of complete DMEM-10 (final dilution series 40-fold to 9,720-fold). The initial dilutions utilized in testing are shown in Table S1.

Plates were fixed by adding 25 µL of 4% formaldehyde/DPBS to the media and incubated for 60 min at room temperature. Plates were washed three times with 150 µL PBS to remove formaldehyde. Plates were then treated with rabbit polyclonal anti-VACV antibody (ViroStat, 8101) diluted 1,000-fold in FFA stain (1× PBS, 1 mg/mL saponin, and 0.1% BSA) in a total volume of 50 µL per well. Plates were incubated at room temperature for 2 h and then washed three times with 150 µL FFA wash buffer (0.05% Triton-X/PBS) by incubating plates with wash for 2 min, then discarding the wash buffer. Plates were then treated with goat anti-rabbit IgG (Bethyl Laboratories, A120-201P) diluted 7,000-fold in 1× FFA stain in a total volume of 50 µL per well. Plates were incubated at room temperature for 1 h in the dark and then washed with 150 µL FFA wash buffer as described above. Plates were stained with 50 µL 0.22 µm-filtered TrueBlue (SeraCare, 5510-0050) for exactly 15 min, then washed twice with 150 µL Milli-Q water. Plates were dried, then counted with CTL BioSpot 7.0.38.7 imager.

### Meso scale discovery V-PLEX multispot orthopoxvirus serology assay

MPXV antibody titers were measured using the MSD V-PLEX Orthopoxvirus Panel 1 (IgG) Kit (MSD, K15688U-2), an electrochemiluminescence assay that quantitatively detects IgG antibodies against five MPXV viral proteins (A29L, E8L, M1R, A35R, and B6R) and their corresponding VACV homologs (A27L, D8L, L1R, A33R, and B5R) in human serum samples. Assay plates were first blocked by adding 150 µL of MSD Blocker A to each well, followed by incubation for at least 30 min with orbital shaking at 700 rpm (JitterBug, Model 270400). During the blocking step, serum specimens were diluted between 1:500 and 1:400,000 in MSD Diluent-100 (MSD, R50AA-2) in 1 mL polypropylene deep-well plates (USA Scientific, 1896-1110). A calibrator series was prepared from a 10× calibrator stock (MSD, C0686-2) by first diluting 10-fold, then serially 4-fold over seven dilutions (CAL-01 through CAL-07). A blank control (CAL-08) was prepared using Diluent-100 alone. Following blocking with Blocker A, the assay plates were washed three times with 300 µL/well of 1× MSD Wash Buffer using an Agilent BioTek plate washer (Model 50TS9V-SN). Following the washing steps, 50 µL of diluted samples, Orthopoxvirus Serology Controls 1 through 3 (MSD, C4686-1), and calibrators were added to the assay plate. Plates were incubated for 2 h at room temperature with orbital shaking at 700 rpm. After incubation, the plates were washed again as described above. Next, 50 µL of SULFO-TAG Anti-Human IgG Antibody (MSD, D21ADF-3) was added to each well, followed by incubation for 1 h at room temperature with shaking at 700 rpm. After the antibody incubation, the plates were washed again as described above, and 150 µL/well of MSD GOLD Read Buffer B was added. The plates were read using the MESO QuickPlex SW 120MM instrument. A detailed description of the MSD assay validation is provided in the Supplementary Material.

### Analysis and statistical methods

Antibody levels and MVA neutralization titers were log-transformed prior to analysis. For the MVA neutralization assay, the ND50 was estimated by four-parameter logistic modeling using the dr4PL R package (version 2.0.0) (20). Antibody levels in AU/mL were determined using the MSD Discovery Workbench Desktop Analysis software (version 4.0). ND50 and MSD estimates that fell below the LLoQ were retained as point estimates derived from curve fitting, rather than being imputed (e.g., as ½ LLoQ). One specimen, P61, had an undetected result for VACV B5R and was set to an AU/mL of ½ LoD. Pearson’s correlation coefficients were calculated to assess relationships between antibody levels across ortholog pairs, while Spearman’s correlation coefficients were used to evaluate associations between antibody levels and MVA neutralization titers. Both correlation coefficients and associated *P*-values were computed using the cor.test function from the base stats package in R (version 4.5.1). Group comparisons of antibody levels, log₂-transformed ortholog pair ratios, and MVA neutralization titers were performed using two-sided t-tests without assuming equal variance. To account for multiple comparisons, *P*-values were adjusted using the Bonferroni correction. The rstatix R package (version 0.7.2) was used to perform t-tests and apply *P*-value corrections (21). Receiver operating characteristic (ROC) curve analysis was conducted using the pROC R package (version 1.19.0.1) (22). For the ROC analysis, baseline sera collected in the longitudinal vaccine group were included in the exposed group if they had a history of vaccination (6 of 22); otherwise, they were allocated to the negative group. All analysis code and figure generation scripts are available at https://github.com/greninger-lab/MPXV_MSD_validation_paper.git. The complete data set is available in Table S1.

## RESULTS

### Validation of MSD orthopoxvirus antibody titer assay

**Fig 1:**
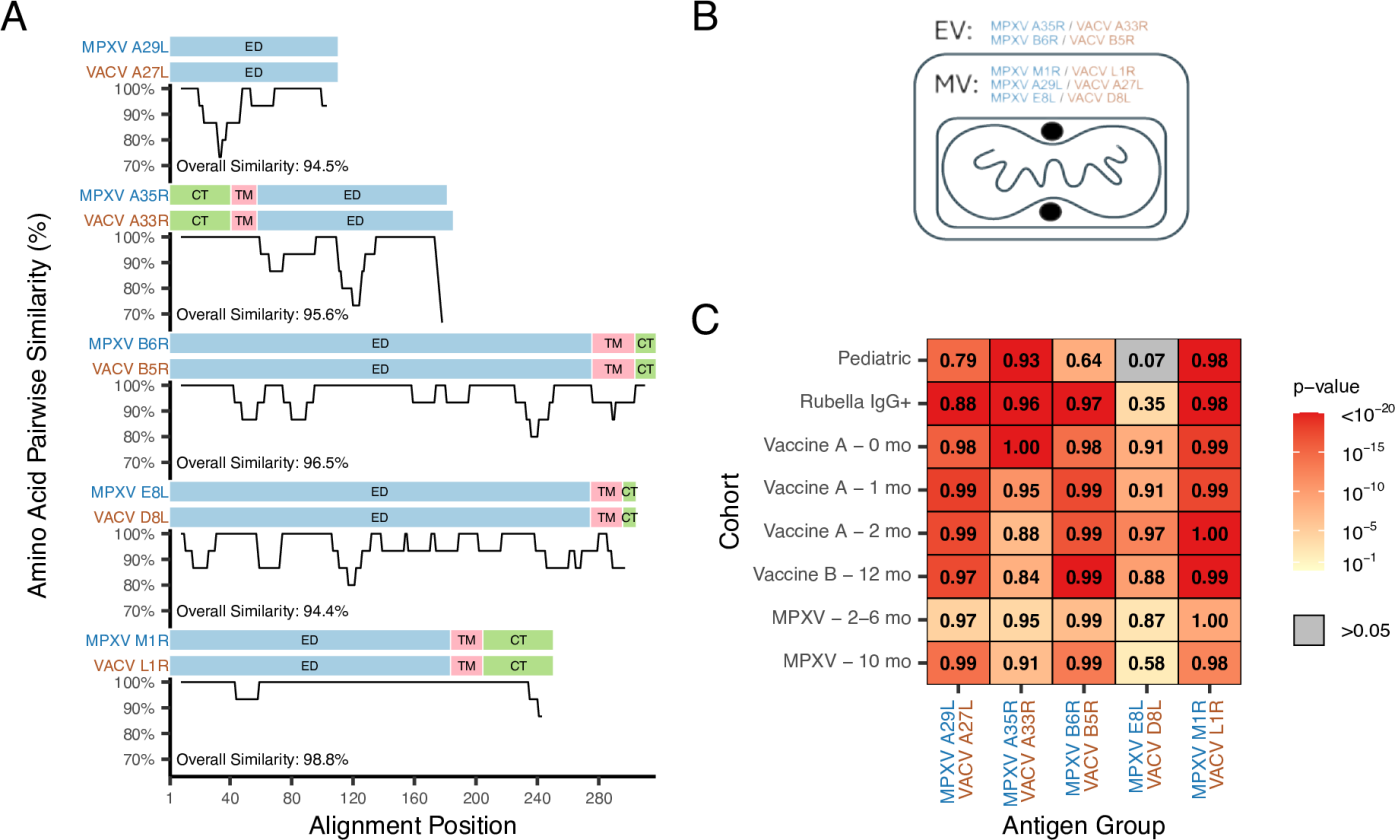
Responses to paired MPXV and VACV antigen orthologs are highly correlated in each cohort. (**A**) Paired MPXV and VACV antigen orthologs in the MSD orthopoxvirus antibody assay are highly conserved. Amino acid similarity is depicted for each ortholog pair using a 15-amino acid sliding window. Endodomain/cytoplasmic tail (CT), TM, and ectodomain (ED) regions are shown above each similarity graph. (**B**) Orthologs selected for the MSD assay are found either on the intracellular MV or extracellular virion (EV). (**C**) Pearson correlations of the antibody level for each paired MPXV and VACV ortholog are shown for each of the specimen cohorts examined. To determine whether a correlation is significantly different from zero (i.e., no correlation), a t-test was performed, and the heatmap is colored by the associated *P*-value of this test, as depicted in the legend key. The P61 VACV B5R result was omitted from the analysis, since the antibody level was undetected.

The MSD V-PLEX Orthopoxvirus Panel 1 (IgG) assay quantifies antibody levels (in units of AU/mL) against five MPXV antigens (A29L, A35R, B6R, E8L, and M1R) and their VACV orthologs (A27L, A33R, B5R, D8L, and L1R). These orthologs are highly conserved (>94% amino acid identity) and presumed to share functional properties (Fig. 1A). VACV A33R and B5R are transmembrane (TM) proteins associated with the enveloped virion (EV) (23–25), while VACV D8L and VACV L1R are intracellular TM proteins associated with the mature virion (MV) (26, 27) (Fig. 1B). VACV A27L is peripherally associated with MV via non-covalent interactions (28).

Our validation of the MSD assay, following Good Clinical Laboratory Practices (GCLP) guidelines (Supplementary Material), established the analytical sensitivity of the assay (Table S2), confirmed acceptable performance for the calibrators and calibrator model (calibrator regression fits *R*^2^ > 0.9; Fig. S3 and Table S3), linearity (slope 0.9–1.0, *R*² > 0.9, intercept < 0.2 AU/mL; Fig. S4), precision (within-lab geometric coefficient of variation (GCV) < 37%; Fig. S5 and S6), accuracy (serology control 1 and 2%RE within ±30% and control 3 < 0.2 AU/mL; Fig. S7), and freeze-thaw robustness (0.5- to 1.5-fold variation over three freeze-thaws; Fig. S8). Minor deviations (e.g., elevated CAL-08 signal for M1R/L1R) did not affect overall performance (Fig. S1). The assay was deemed suitable for quantitative serology and subsequent cohort testing.

### Anti-orthopoxvirus IgG antibody levels are elevated in sera from vaccinated and MPXV-infected individuals

We assessed serological responses to MPXV infection and vaccination on the MSD assay across six cohorts, including two negative cohorts, two JYNNEOS-vaccinated cohorts, and two MPXV-infected cohorts (see Materials and Methods). Antibody responses to orthologs were strongly correlated across exposed groups (⍴ > 0.8 for most comparisons; Fig. 1C), consistent with sequence conservation. MPXV E8L and VACV D8L exhibited weaker correlations, especially among presumed negative cohorts and MPXV-infected individuals due to distinct responses to the ortholog pairs (Fig. S9).

**Fig 2:**
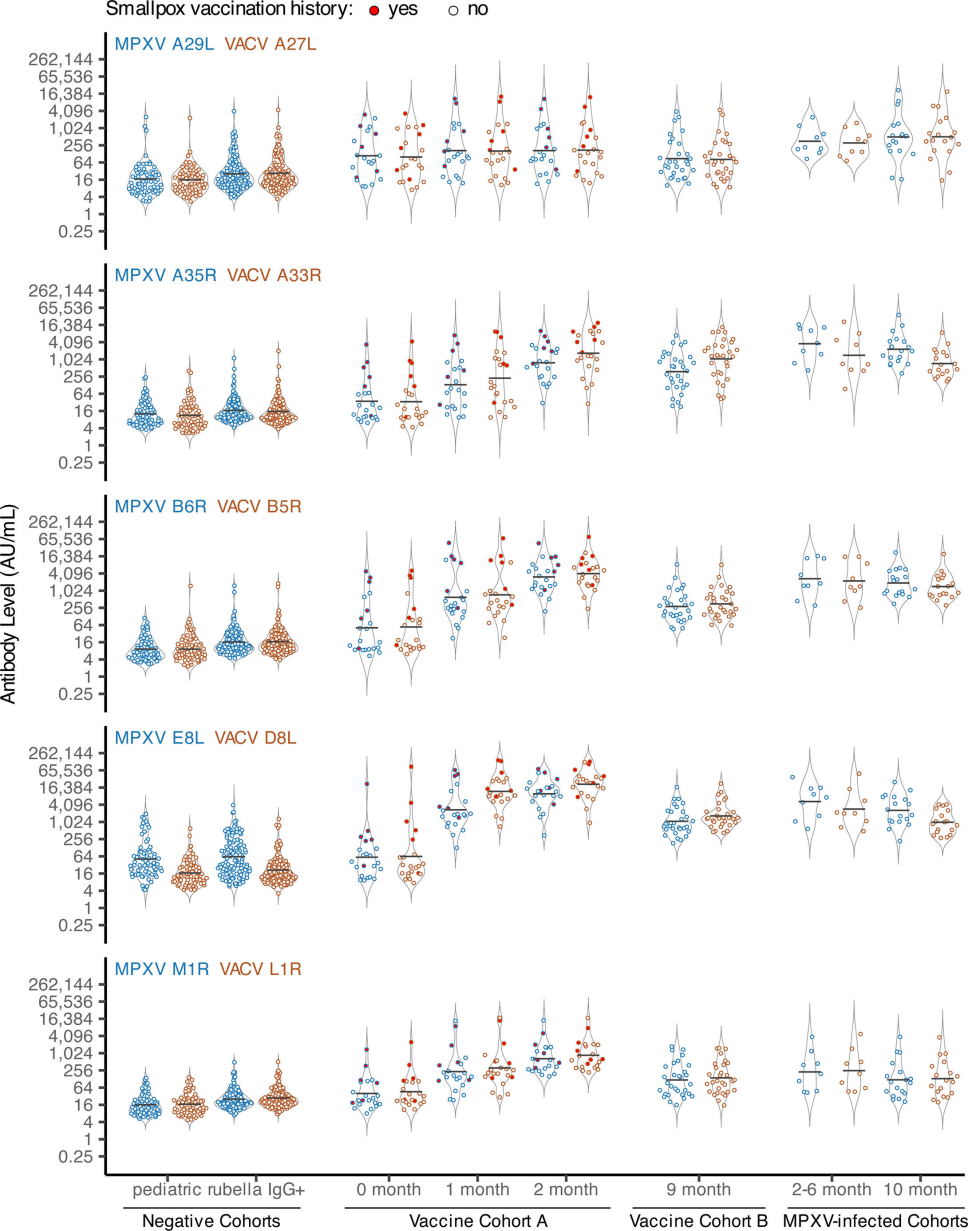
Anti-orthopoxvirus IgG antibody levels are elevated in vaccinated or MPXV-infected individuals. Eight groups of specimens from six cohorts (x-axis) were tested using the MSD assay, and signal values were converted to AU/mL. Responses to MPXV orthologs are shown in blue, and VACV orthologs in brown. The ortholog pair for each plot is indicated in the upper left corner. Black lines represent group geometric mean titers (GMT), and untrimmed violin plots show the distribution behind individual data points. The points corresponding to individuals with a known history of smallpox vaccination are filled in with red.

We next compared antibody responses across cohorts for each ortholog pair (Fig. 2) and evaluated the statistical significance of observed differences (Table S4, Fig. S10).

Negative cohort GMTs were generally low, though antibody levels overlapped with the exposed cohorts. Adult rubella IgG+ specimens tended to have slightly higher GMTs compared to the pediatric cohort (e.g., VACV L1R: Pediatric 16.7 AU/mL vs. Rubella IgG+ 27.7 AU/mL; *P*-value < 0.05). Within the negative cohorts, GMTs were similar across ortholog pairs, except for MPXV E8L/VACV D8L (e.g., Pediatric: MPXV E8L 51.4 AU/mL vs. VACV D8L 16.7 AU/mL; *P*-value < 0.05).

Baseline GMTs in the Vaccine A cohort trended higher relative to the negative cohorts. When individuals with a known history of smallpox vaccination were removed (see red-filled points, Fig. 2), Vaccine A baseline GMTs were similar to the negative cohorts, except for MPXV A29L/VACV A27L (data not shown). Post-vaccination GMTs for Vaccine A and B cohorts were generally higher than negatives and Vaccine A baseline (0 month), except for MPXV A29L/VACV A27L, which had modest increases that were significant only when compared to the Pediatric cohort (e.g., MPXV A29L: Pediatric vs. Vaccine A-1 month, 16.9 vs. 166.4 AU/mL, *P*-value < 0.05; MPXV A29L: Vaccine cohort A-0 month vs. Vaccine cohort A-1 month, 110.0 vs. 166.4 AU/mL, *P*-value > 0.05). Within the Vaccine A cohort, GMTs increased from baseline to 2 months post-vaccination (e.g., MPXV E8L: Vaccine A, 1 month vs. 2 months, 2,710.5 vs. 9,849.5 AU/mL, *P*-value > 0.05), except for MPXV A29L/VACV A27L. In Vaccine B, GMTs at 10 months post-vaccination were significantly lower than those in Vaccine A at 2 months (e.g., MPXV B6R: Vaccine B-9 months vs. Vaccine A-2 months, 285.0 vs. 3,099.1 AU/mL; *P*-value < 0.05), consistent with waning antibody responses.

Across all ortholog pairs, MPXV-infected cohorts had significantly higher GMTs than negatives (*P*-value < 0.05, with limited exceptions within M1R/L1R). Unlike in the vaccinated cohorts, MPXV-infected cohorts showed significantly higher MPXV A29L/VACV A27L titers relative to negatives (*P*-value < 0.05).

### Antibody level ratios between orthologs reveal distinct responses in infected vs. vaccinated cohorts

**Fig 3:**
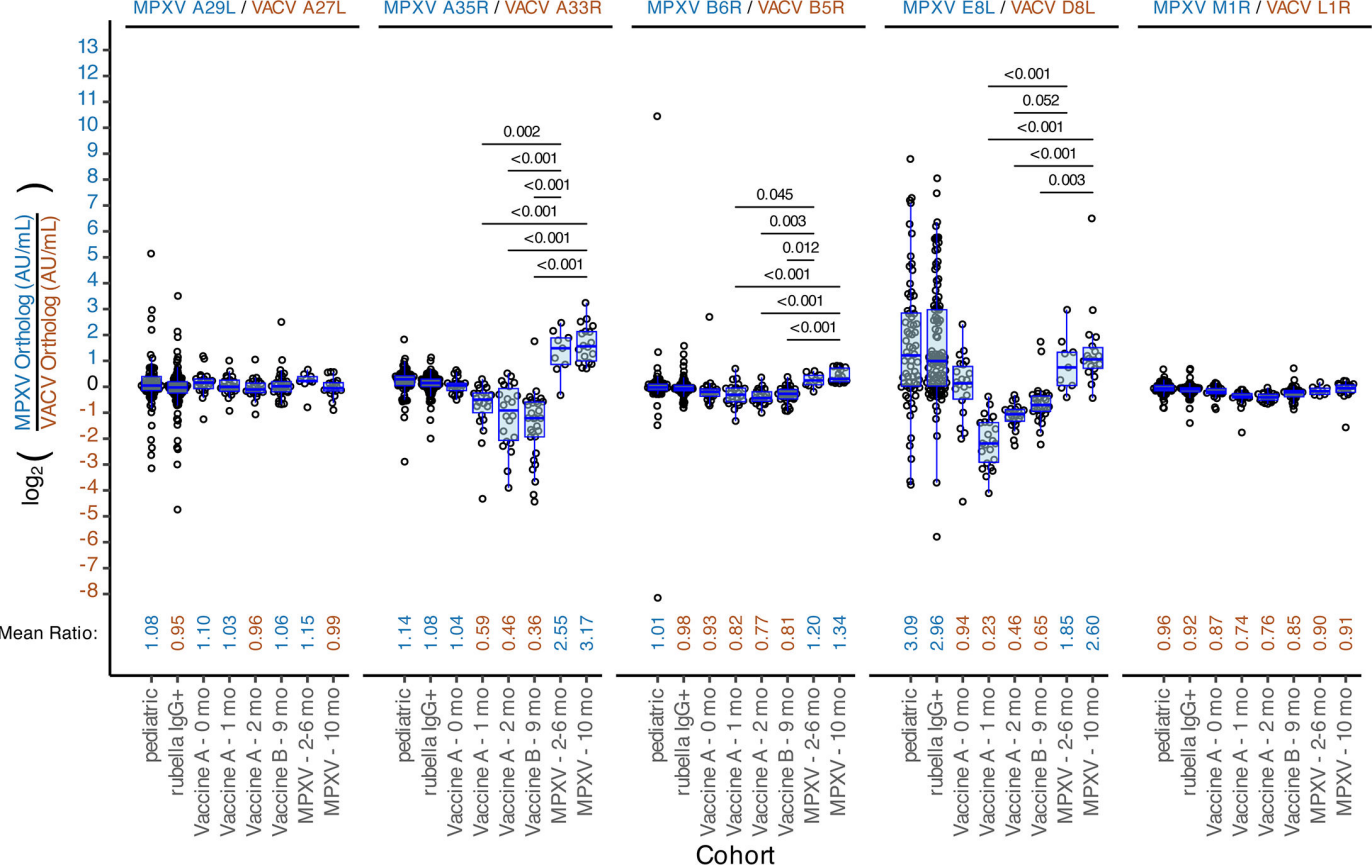
The response ratio between MPXV and VACV orthologs was significantly different for several ortholog antigen pairs. For each result, the ratio of the AU/mL result of the MPXV ortholog over the VACV ortholog was calculated, log_2_-transformed, and plotted for each ortholog pair and cohort. The y-axis labels have been colored such that positive-log-fold changes are blue (MPXV > VACV ortholog response) and negative-log-fold changes are brown (MPXV < VACV ortholog response). Overlaid is a box plot with the central line marking the median value, the hinges indicating the first and third quartile or interquartile range (IQR), and the whiskers extending to the highest or lowest value within 1.5 times the IQR range. The mean ratio for each cohort was calculated and displayed at the bottom of each graph. Mean ratio values are colored such that responses that are stronger to the MPXV ortholog are colored blue and responses that are stronger to the VACV ortholog are colored brown. Utilizing the mean ratio values, we performed a t-test comparing ratios between cohorts using a Bonferroni correction for multiple comparisons. Significant t-test (*P*-value < 0.05) results are shown in the upper part of each graph, only for the comparisons between the MPXV-infected and MVA-BN vaccinated; see Table S5 for all comparisons. The horizontal black bar indicates the groups being compared for a given *P*-value.

To investigate differences in antibody responses between MPXV and VACV orthologs, we calculated the ratio of MPXV to VACV antibody responses for each ortholog pair and compared mean ratios between cohorts using two-sided t-tests (Fig. 3 and Table S5). For MPXV A29L/VACV A27L and MPXV M1R/VACV L1R, mean ratios did not differ significantly between exposed groups. In contrast, significant differences in MPXV/VACV response ratios were observed for MPXV A35R/VACV A33R, MPXV B6R/VACV B5R, and MPXV E8L/VACV D8L, with MPXV-infected cohorts consistently showing higher MPXV/VACV ratios, while vaccinated cohorts showed lower MPXV/VACV ratios. For example, the MPXV A35R/VACV A33R mean ratio was significantly elevated in the MPXV-infected cohort at 2–6 months post-infection compared to vaccinated cohorts at 1, 2, and 9 months (2.55 vs. 0.59, 0.46, and 0.36, respectively; *P*-values < 0.01). The decline in MPXV A35R/VACV A33R ratio in vaccinated cohorts suggests a shift toward relatively higher VACV A33R responses longitudinally. Conversely, the MPXV E8L/VACV D8L ratio increased over time in vaccinated cohorts at 1, 2, and 9 months (0.23, 0.46, and 0.65, respectively), indicating a relative increase in MPXV E8L reactivity. Finally, the Pediatric and Rubella IgG+ cohorts exhibited elevated MPXV E8L/VACV D8L ratios (3.09 and 2.96, respectively), driven mostly by higher MPXV E8L responses, potentially reflecting non-specific binding to this antigen.

### Sera from vaccinated or MPXV-infected individuals were neutralized against MVA

**Fig 4:**
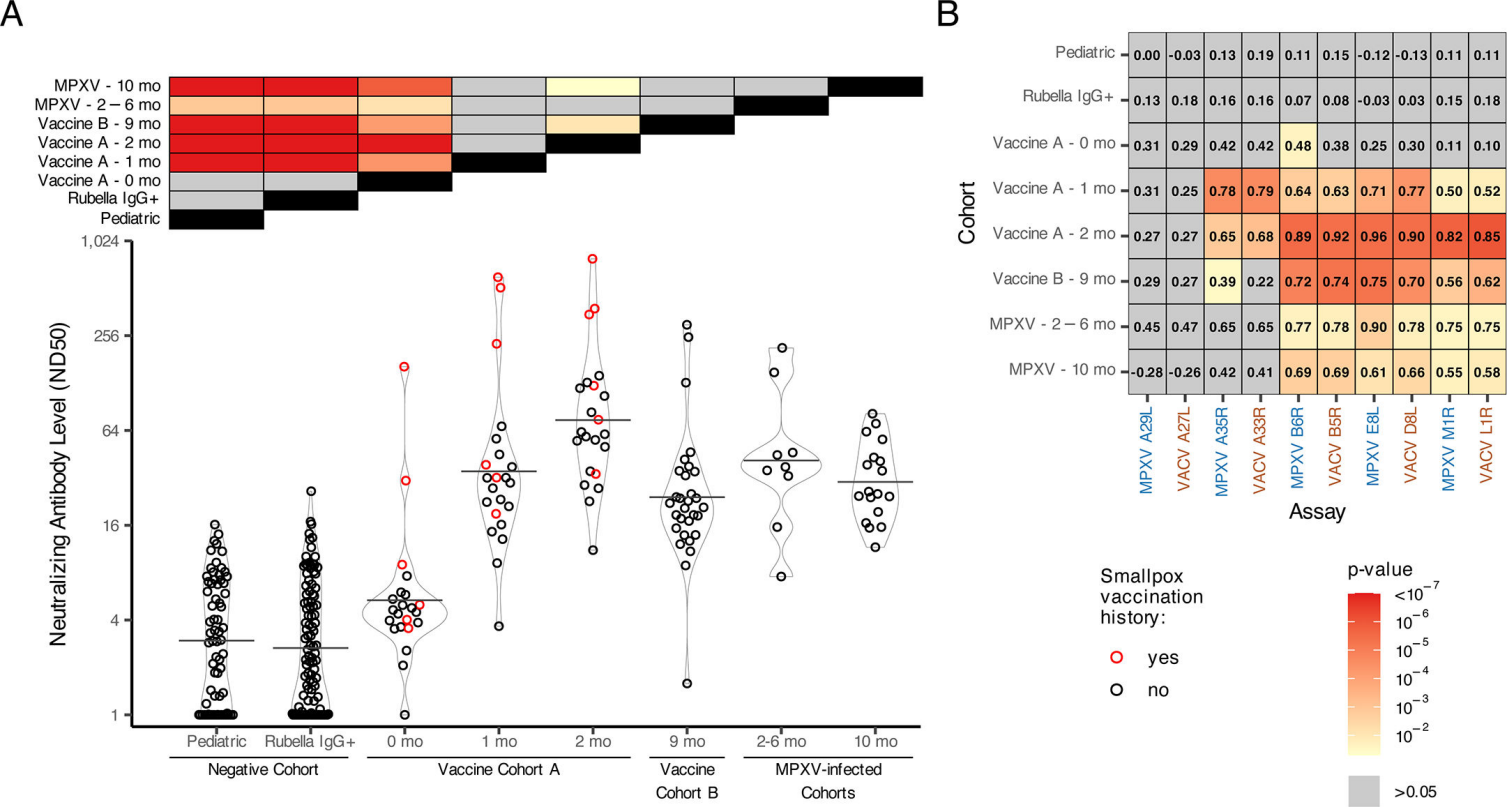
Sera from vaccinated (post-baseline) or MPXV-infected exhibited low-level neutralization of Vaccinia MVA, which was generally correlated with anti-orthopoxvirus antibody level. (**A**) The ND50 value against MVA was determined for all the sera tested in the MSD orthopoxvirus antibody titer assay and plotted by cohort. Prior smallpox vaccination history is indicated by red-filled points. The black horizontal lines correspond to each cohort's GMT. Two-sided t-tests were used to compare ND50 titers between groups, and Bonferroni-adjusted *P*-values were visualized in a heatmap along the top of the figure with yellow-to-red shading indicating statistical significance (*P*-value < 0.05). (**B**) Spearman’s correlations were calculated between antibody titer results for each antigen and the MVA neutralization ND50 results within each cohort separately. To determine whether a correlation is significantly different from zero (i.e., no correlation), a t-test was performed, and the fill value in each tile corresponds to the *P*-value of this test as shown in the legend key.

To assess the correlation between anti-orthopoxvirus antibody levels determined by the MSD assay and functional neutralizing activity, we performed a focus reduction neutralization test (FRNT) using MVA to quantify ND50 titers in all cohorts (Fig. 4A). Two-sided t-tests were used to compare ND50 titers between groups. Overall, ND50 titers were low across cohorts, consistent with previous reports of orthopoxvirus neutralization assays that lack complement (29). The negative and Vaccine A baseline cohorts exhibited minimal neutralizing activity (ND50 < 20), with a few exceptions: one Rubella IgG+ specimen (RU6, ND50 = 26.3) and two Vaccine A baseline specimens from individuals with a history of smallpox vaccination (VRC30, ND50 = 162.6; VRC26, ND50 = 30.7; Table S1). The higher ND50 GMT of the Vaccine A baseline group, relative to the negative cohorts, in part reflects prior smallpox vaccination in some individuals (Fig. 4A, red colored points). Following vaccination, Vaccine A participants showed a marked increase in ND50 GMTs: a 6.6-fold rise from baseline to 1 month (ND50 GMT 5.3 to 35.2), and a further 2.1-fold increase from 1 to 2 months (ND50 GMT 35.2 to 74.4). Relative to Vaccine A cohort participants at 2 months post-vaccination, Vaccine B cohort participants (9 months post-vaccination) had 3.1-fold lower ND50 GMTs (ND50 GMT 24.1 vs. 74.4), again consistent with waning neutralizing titers. Among MPXV-infected individuals, ND50 GMTs were comparable between the 2-6 month and 10-month post-infection groups (ND50 GMT 41.2 vs. 30.1), and similar to titers observed in Vaccine A (1 month) and Vaccine B (9 months) cohorts. The highest neutralizing responses were observed in Vaccine A cohort individuals with prior smallpox vaccination.

We next compared MSD-derived antibody levels with MVA FRNT ND50 values across all antigens and cohorts (Fig. 4B; Fig. S11). In the negative cohorts (Pediatric and Rubella IgG+), binding titers and neutralization were poorly correlated (Spearman ⍴ < 0.5), with individual responses randomly distributed between antibody level and ND50 (Fig. 4B; Fig. S11). A similar pattern was observed in the Vaccine A baseline cohort, except for 2–3 individuals with prior smallpox vaccination, who showed stronger correlation between ND50 and binding titers (Fig. S11, red-filled points). In the Vaccine A cohort, the correlation between antibody titer and MVA neutralization strengthened over time following vaccination for all antigens except MPXV A29L/VACV A27L and MPXV A35R/VACV A33R. Neutralizing titers post-vaccination were most strongly correlated with antibody binding levels to MPXV B6R/VACV B5R, MPXV E8L/VACV D8L, and MPXV M1R/VACV L1R. Interestingly, the correlation between MPXV A35R/VACV A33R and MVA neutralization appeared strongest 1 month post-vaccination but declined at 2 months post-vaccination. MPXV-infected individuals showed a similar antigen-specific correlation pattern to post-baseline-vaccinated cohorts.

### Diagnostic performance of the MSD assay using single antigens or ortholog pair response ratios

To evaluate whether antibody levels for individual antigens can distinguish people with prior mpox from negative controls (Rubella IgG+ and Pediatric) or vaccinated from negative controls, we performed ROC analysis. Starting with MPXV-infected versus negative controls, area under the curve (AUC) values ranged from 0.892 to 0.998, with MPXV A35R and MPXV B6R showing the highest performance (Fig. S12, Table S6). Youden index thresholds determined for MPXV A35R and MPXV B6R were 295.6 AU/mL and 252.1 AU/mL, respectively. At these thresholds, each antigen had a specificity of 1.000 (95% CI 1.000–1.000) and sensitivity of 0.990 (95% CI 0.974–1.000) for MPXV A35R and 0.979 (95% CI 0.959–0.995) for MPXV B6R. The VACV orthologs of these antigens, VACV A33R and VACV B5R, showed similar performance with sensitivity/specificity > 0.95. MPXV M1R and VACV L1R exhibited the lowest AUCs (0.895 and 0.891, respectively) and specificity/sensitivity < 0.9. In the comparison of vaccinated versus negative controls, AUC values ranged from 0.785 to 0.991, with VACV D8L showing the highest performance (Fig. S2, Table S7). VACV D8L achieved a specificity of 0.988 (95% CI 0.963–1.000) and sensitivity of 0.969 (95% CI 0.949–0.995) at 233.1 AU/mL. Its MPXV ortholog, E8L, performed less well (AUC 0.946), likely due to broader antibody level distributions in the negative cohorts (Fig. 2; Fig. S13). MPXV A29L and VACV A27L exhibited the lowest AUCs (0.792 and 0.785, respectively) and specificity/sensitivity < 0.9.

**Fig 5:**
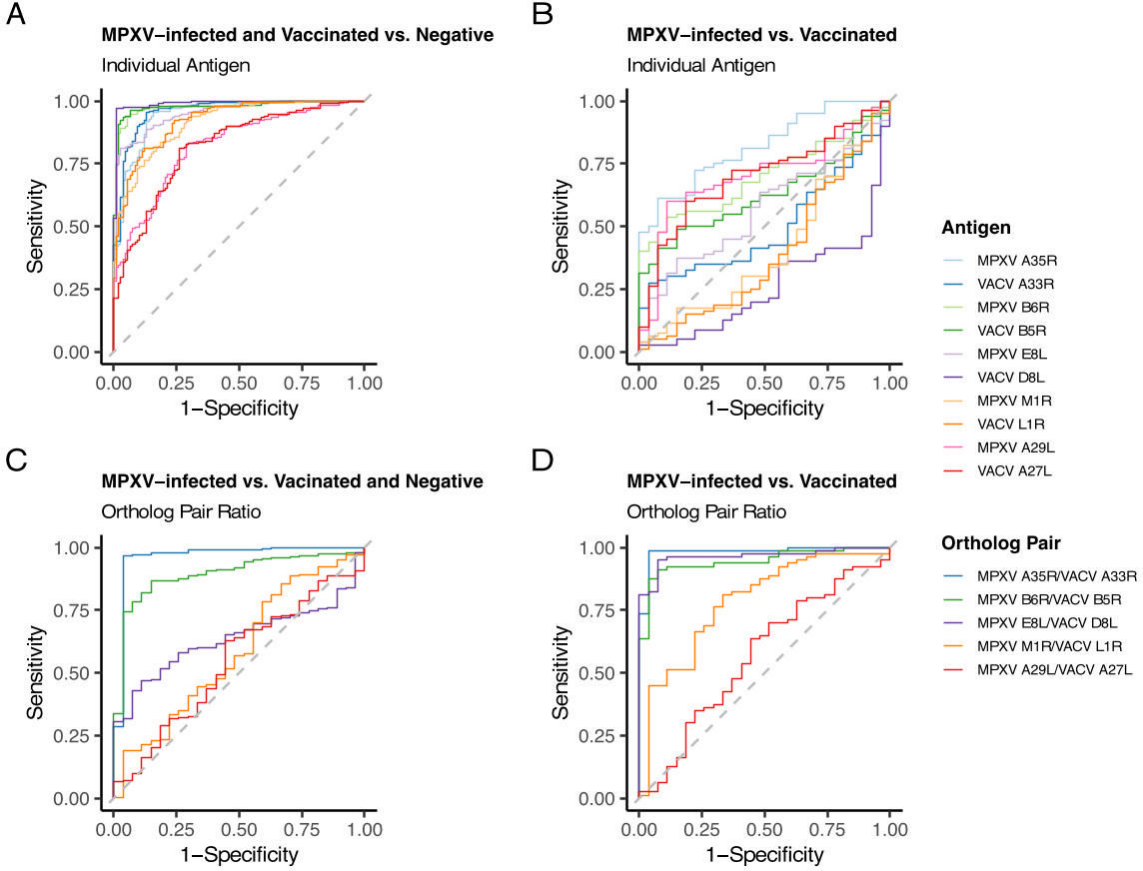
ROC curves utilizing either the anti-orthopoxvirus antibody level results or the ratio of antibody level to ortholog pairs. MPXV-infected refers to all sera from known MPXV-infected individuals, Vaccinated refers to all sera from known vaccinated individuals, and Negative refers to sera from the rubella and pediatric negative cohorts combined. Baseline sera collected prior to vaccination (Vaccine A-0 month) were included in the Vaccinated group if they had a history of smallpox vaccination; otherwise, they were categorized as negative (6 out of 22 individuals had a previous history of vaccination). (**A**) ROC curves for all individual antigens were generated by comparing all known MPXV-infected or Vaccinated (*n* = 107) to all Negative (*n* = 195). (**B**) ROC curves for all individual antigens were generated by comparing all known MPXV-infected (*n* = 27) to all known Vaccinated (*n* = 80). (**C**) ROC curves for all antigen ortholog pairs generated by comparing antigen ortholog pair ratios from known MPXV-infected (*n* = 27) to Vaccinated and all Negative (*n* = 275). (**D**) ROC curves for all antigen ortholog pairs generated by comparing antigen ortholog pair ratios from known MPXV-infected (*n* = 27) to known Vaccinated (*n* = 80). Summary statistics from this analysis can be found in Table S8 through S11.

We also evaluated the performance of the assay to distinguish exposed (MPXV-infected and vaccinated combined) from negative controls (Rubella IgG+ and pediatric). AUC values ranged from 0.818 to 0.991, with VACV D8L showing the highest performance (Fig. 5A; Table S8). VACV D8L achieved a specificity of 0.991 (95% CI 0.972–1.000) and sensitivity of 0.969 (95% CI 0.944–0.990) at 233.1 AU/mL (Table S8). As with the vaccinated vs. negative control comparison, the MPXV E8L ortholog performed less well (AUC 0.953).

Next, ROC analysis was performed to evaluate the performance of the assay to distinguish specimens from MPXV-infected from vaccinated individuals. Here, lower AUCs (0.260–0.819) were observed, with MPXV A35R performing best (AUC 0.819; Fig. 5B and Table S9). However, sensitivity was limited (0.613 at 621.1 AU/mL), reflecting the challenge of differentiating infection from vaccination using single-antigen thresholds.

Based on Youden index thresholds determined on differentiating specimens from exposed or negative individuals (Fig. 5A and Table S8), 60% (41/68) of Pediatric and 64% (71/111) of Rubella IgG+ specimens exceeded the cutoff for at least one antigen (Fig. S13). Two Rubella IgG+ specimens exceeded thresholds for all ten antigens and were MVA neutralizing (RU6, ND50 26.3; RU58, ND50 13.3; Table S13), suggesting prior orthopoxvirus exposure or vaccination. Other specimens exceeded fewer thresholds and lacked neutralization, indicating possible cross-reactivity.

Since we identified that certain ortholog pairs showed differential responses between vaccinated and MPXV-infected individuals (Fig. 3), we next assessed the discriminatory power of the ortholog pair ratios. We generated ROC curves comparing MPXV-infected specimens to vaccinated and negative cohorts (Fig. 5C and Table S10). MPXV A35R/VACV A33R and MPXV B6R/VACV B5R ratios performed best, with AUCs of 0.965 and 0.894, respectively. For MPXV A35R/VACV A33R, the optimal threshold was a ratio of 1.6, yielding 0.963 specificity (95% CI: 0.889–1.000) and 0.967 sensitivity (95% CI: 0.945–0.989). MPXV E8L/VACV D8L had poor performance when negatives were included (AUC 0.639) but improved when negatives were excluded with an AUC 0.965, specificity 0.926 (95% CI 0.852–1.000), sensitivity of 0.950 (95% CI 0.812–1.000; Fig. 5D and Table S11). In addition, when negatives were excluded, MPXV A35R/VACV A33R and MPXV B6R/VACV B5R ortholog ratios AUCs increased from 0.965 to 0.983 and 0.894 to 0.946, respectively.

## DISCUSSION

Here, we show results from validation and testing of a commercial MSD multiplex antibody assay, confirming it meets GCLP criteria. To assess clinical performance characteristics, sera were tested in the MSD assay from presumed unexposed individuals, vaccinated individuals, and individuals with prior mpox. Both vaccination and MPXV infection induced measurable antibody responses to all MPXV and VACV antigens in the MSD assay, but vaccination and infection cohorts differed in the magnitude and pattern of responses. Antibody levels correlated with MVA-neutralization for most ortholog pairs, further corroborating assay performance. Finally, ROC analysis demonstrated that while single antigens could distinguish exposed from unexposed individuals, ortholog response ratios more effectively differentiated infection from vaccination.

Testing of these cohorts revealed some expected patterns. Antibody levels to MPXV A29L/VACV A27L showed little change post-vaccination, consistent with poor induction by third-generation smallpox vaccines (Fig. 2) (14, 17, 30–33). In contrast to these results, older attenuated vaccine strains have been observed to elicit stronger VACV A27L responses (32, 34). Review of published MVA and MVA-BN genome sequences indicates that the VACV A27L ORF is intact (GenBank: U94848 and DQ983238, respectively), suggesting that further attenuation of the third-generation vaccine strains may have impaired A27L immunogenicity. In addition, even when VACV A27L responses are induced by vaccination, they have not been found to constitute a major fraction of neutralization activity (34, 35). Although not a longitudinal sampling, at 9 months post-vaccination (Vaccine B), antibody GMTs to all antigens were generally lower than those observed at 2 months post-vaccination (Vaccine A), aligning with reports of waning antibody levels post-vaccination (36, 37) (Fig. 2). Among single antigens, VACV D8L, VACV B5R, MPXV B6R, VACV A33R, and MPXV A35R best distinguished individuals exposed by either natural MPXV infection or vaccination, consistent with prior MSD assay studies (13, 17). When focusing on MPXV-infected-only versus negative controls, MPXV A35R and MPXV B6R performed best, and when focusing on vaccinated-only versus negative controls, VACV D8L performed best. Custom MSD plates (38) and multiplex bead-based platforms (14, 16) that utilized an overlapping but distinct set of antigens also generally identified these antigens as discriminatory, except MPXV A35R did not perform as well in the custom MSD assay (38). We also observed that MPXV M1R/VACV L1R antigens showed better sensitivity and specificity in distinguishing specimens from vaccinated individuals from negative controls compared to the MPXV-infected specimens, consistent with stronger MPXV M1R/VACV L1R responses in the vaccinated relative to the MPXV-infected (Fig. 2) (15, 17). Likewise, the opposite dynamic was observed for the MPXV A29L/VACV A27L ortholog pair due to weaker responses among the vaccinated (Fig. 2) (17). However, overall, MPXV M1R/VACV L1R and MPXV A29L/VACV A27L, consistent with other studies, were generally poor or inconsistent markers (13, 14, 16, 17, 38).

Although antigens included in the MSD assay generally distinguished exposed from unexposed individuals, no single antigen separated MPXV-infected from vaccinated (Fig. 5B). Prior strategies include targeting antigens absent in MVA-BN, such as MPXV A27L (14, 15), MPXV A26L (38), and MPXV B21R (7, 12, 16) or those with exposure-dependent responses (not ortholog dependent), such as MPXV A29L/VACV A27L (elevated in MPXV-infected) and MPXV M1R/VACV L1R (elevated in vaccinated) (14, 15, 17). Although the MSD assay includes these latter two sets of orthologs and we observed exposure-dependent responses (Fig. 2), these orthologs did not appear to perform well in distinguishing MPXV-infected from vaccinated in our data (Fig. 5B and Table S9). Differential ortholog ratios have been reported elsewhere (14–17, 39), and one group combined the MPXV B6R/VACV B5R ratio and MPXV A27L to distinguish MPXV-infected from vaccinated (38). In this study, MPXV A35R/VACV A33R, MPXV B6R/VACV B5R, and MPXV E8L/VACV D8L ratios could discriminate MPXV-infected from vaccinated, with the strongest predictor being MPXV A35R/VACV A33R (Fig. 5C and Table S10). One limitation of this approach is that differential responses may be less obvious when individuals are both vaccinated and MPXV-infected (16). Continued evolution of MPXV surface antigens in future outbreaks may also lead to changing responses between MPXV and VACV orthologs.

Since these antigens are involved in orthopoxvirus neutralization, we further validated the MSD assay by assessing whether antibody levels correlated with MVA neutralization. Significant positive correlations were observed in post-baseline vaccinated and MPXV-infected individuals for all antigens except MPXV A29L/VACV A27L (Fig. 5). A prior study using the same assay reported weaker correlations with neutralization, likely due to differences in the neutralization target (MPXV Clade IIb B.1 or VACV WR instead of MVA) and unknown exposure histories, limiting direct comparison (18).

One challenge of validating orthopoxvirus serological assays is obtaining specimens known to be unexposed to orthopoxvirus either through natural infection or, more often, vaccination. Although the negative cohorts assembled here likely lacked smallpox vaccination since they were born after 1972 and had no known background of orthopoxvirus exposure, two Rubella IgG+ specimens (RU6 and RU58) showed elevated antibody levels and MVA-neutralization, suggesting prior vaccination or exposure, though unconfirmed. Pediatric specimens have a higher likelihood of not having orthopoxvirus exposure and have been used by others to establish threshold values in the MSD assay (17). However, pediatric specimens may not be fully representative of the responses observed in adults. In fact, in this study, Rubella IgG+ GMTs within orthologs consistently trended higher compared to the Pediatric GMTs. Based on thresholds for distinguishing exposed vs. unexposed individuals (Table S8), there were many negative specimens that exceeded these thresholds for a subset of antigens (commonly 1–2 antigens, Fig. S13). Cross-reactive responses are not uncommon in these assays (15, 16, 40). MPXV E8L appears to have significant background or cross-reactive responses in both our negative cohorts (Fig. 2 and Fig. S13). Requiring positivity for greater than one antigen may improve assay performance (14, 16, 17).

There are several limitations to this study. First, we did not have individuals that were both vaccinated and MPXV-infected, which might affect differential response to the MPXV/VACV ortholog pairs, as observed elsewhere (16). The lack of an international standard for antiserum to VACV or MPXV complicates commutability of this work, although the calibrators provided by MSD should allow for harmonization of results between labs utilizing the commercial MSD assay. Our MPXV-infected cohorts were relatively small (a total of 27 specimens), which may impact ROC analysis. We only assessed neutralization against the attenuated MVA strain, and although prior studies report that VACV- and MPXV-based neutralization assays are generally well correlated (29, 41), we cannot exclude that results may differ between these systems. The vaccinated and MPXV-infected cohorts were strongly skewed toward men (77%–100% male) relative to the Rubella IgG+ cohort (13% male) and the Pediatric cohort (51% male), though this adequately reflects the population most at risk of MPXV infection. The robustness of antibody responses and the ability to differentiate between MPXV and VACV responses may vary with factors such as immunocompetence, age, and underlying health status, which were factors not fully addressed in this study. Lastly, though we performed Youden index analysis to determine post hoc thresholds for exploratory purposes, we lack a validation cohort to test whether these thresholds confer the expected sensitivity and specificity.

Overall, we demonstrate that the MSD orthopoxvirus IgG assay meets GCLP-based validation standards and can reliably distinguish orthopoxvirus-exposed from unexposed individuals. Furthermore, differential antibody responses to specific MPXV/VACV ortholog pairs (particularly MPXV A35R/VACV A33R and MPXV B6R/VACV B5R) may be useful for discriminating JYNNEOS-vaccinated from MPXV-infected individuals. Antibody levels measured by the MSD assay were generally correlated with MVA neutralization activity, supporting its relevance as a surrogate marker of the immune response. However, limited discriminatory performance for the MPXV A29L/VACV A27L and MPXV M1R/VACV L1R antigen pairs, along with the elevated background observed for MPXV E8L, highlights areas for further assay refinement. Inclusion of additional antigens, particularly those with divergent immunogenicity between MPXV and VACV or those that are missing in the attenuated vaccine strains, could further improve the assays’ utility to identify prior exposure and differentiate between routes of exposure. Given waning global population immunity to orthopoxviruses, MPXV outbreaks are likely to persist, and robust serological assays will be critical for understanding MPXV infection prevalence, defining immune correlates, and guiding vaccine policy and booster strategies.

## Data Availability

Line-item testing data is available in Table S1.

## SUPPLEMENTAL MATERIAL

Supplemental figures jcm.01548-25-s0001.pdf Figure S1 to S13.

Supplemental text jcm.01548-25-s0002.docx Supplemental material and methods.

Supplemental tables jcm.01548-25-s0003.xlsx Tables S1 to S11.

ASM does not own the copyrights to Supplemental Material that may be linked to, or accessed through, an article. The authors have granted ASM a non-exclusive, world-wide license to publish the Supplemental Material files. Please contact the corresponding author directly for reuse.
