# Supplementary material for: Differentiating mpox infection and vaccination using a validated multiplex orthopoxvirus IgG serology assay
Source: J Clin Microbiol. 2025 Dec 29;64(2):e01548-25. doi: 10.1128/jcm.01548-25 (PMC12892956; doi:10.1128/jcm.01548-25)
Supplement: Supplemental figures — Figure S1 to S13. [file jcm.01548-25-s0001.pdf]

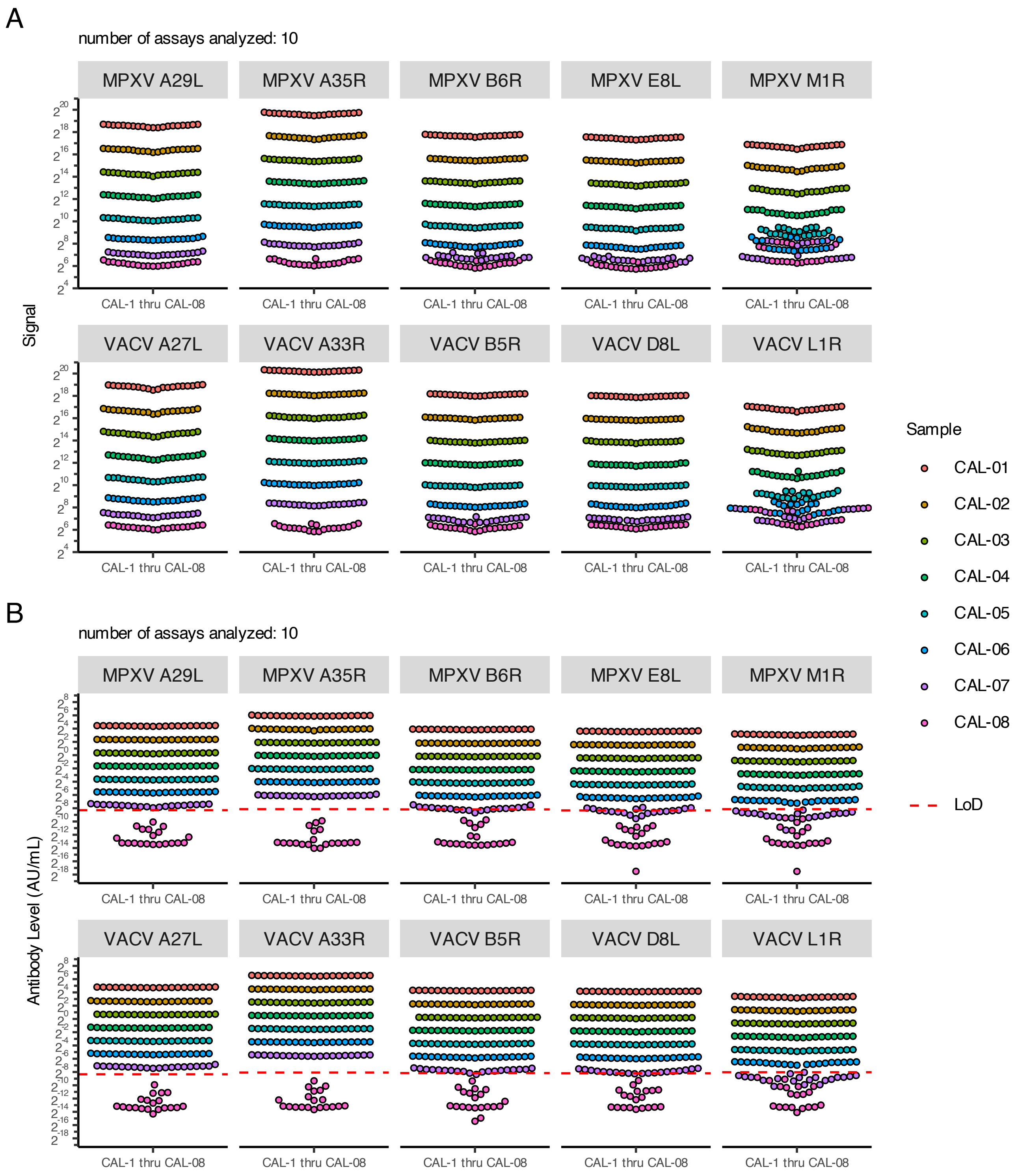

Figure S1: Signal results from different days overlap among the lowest calibrators, but conversion to AU/mL normalizes for the day-to-day differences in Signal. Shown are calibrator results from ten independent runs of the MSD Orthopoxvirus assay in units of Signal (A) and antibody level in AU/mL (B). For MPXV A29L, VACV A27L, MPXV A35R, VACV A33R antigens CAL-01 thru CAL-08 are entirely distinguishable by Signal even between runs on different days. However, the remaining antigens showed some degree of Signal overlap between CAL-05 thru CAL-08. Examination of Signal levels within any given run showed that the Signal levels are distinguishable for CAL-01 thru CAL-07 for each of the antigens. Conversion of Signal to AU/mL normalizes results such that overlap is limited to CAL-07 and CAL-08. The limit of detection (LoD) for each antigen for each run was calculated following the described manufactures recommendation and the average of these results is indicated by the red dashed line. When the CAL-08 signal fell below the lower asymptote of the 4PL model used to convert Signal to AU/mL, an AU/mL value could not be calculated and was arbitrarily set to the lowest reportable value based on the 4PL model fit.

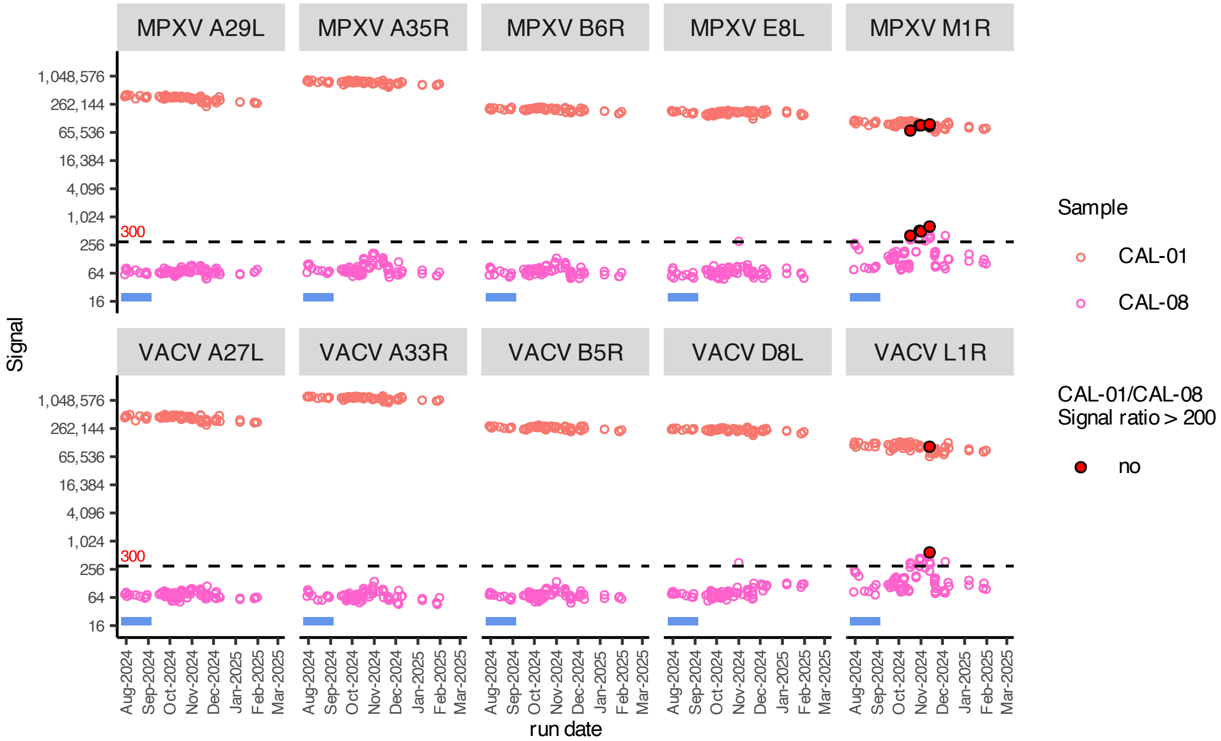

Figure S2: Greater variation in CAL-08 Signal over time is evident for antigens MPXV M1R and VACV L1R. Shown are CAL-01(red) and CAL-08 (purple) Signal values both during validation of the assay (see light-blue shaded bars) and subsequent testing over the following 5-6 months. The dashed blank line indicates the initial quality control threshold that CAL-08 was expected to not exceed. Red filled points are assays that did not meet CAL-01/CAL-08 Signal ratio of at least 200, which included five M1R assays and one L1R assay.

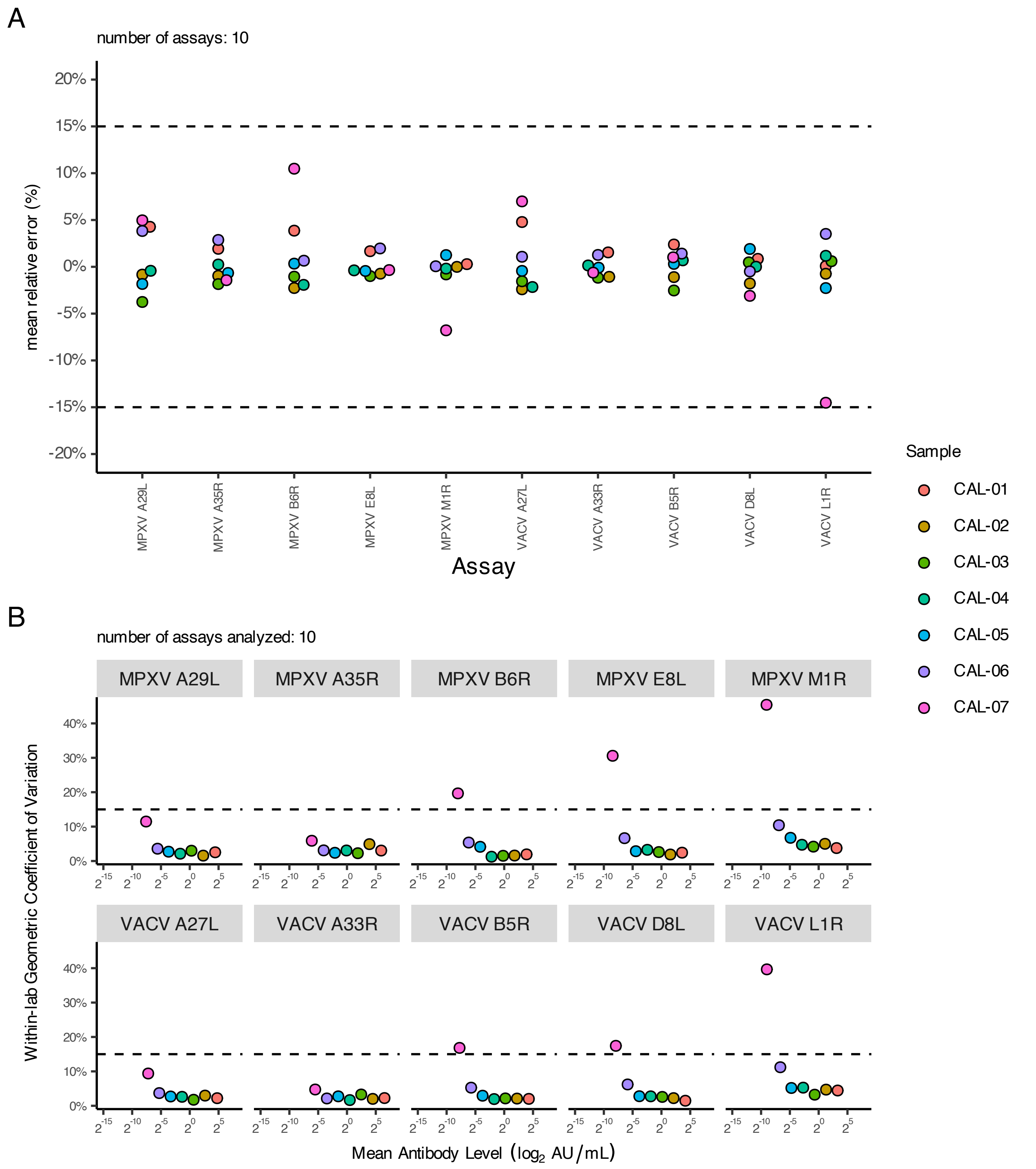

Figure S3: Average relative error of calibrators is within +/- 15 and within-lab geometric coefficient of variation of each calibrator is within 15% for CAL-01 thru CAL-06. Assay calibrators (CAL-01 thru CAL-07) were tested in duplicate over ten runs (A) Shown is the mean percent relative error for each calibrator (CAL-01 thru CAL-07) with the acceptability criteria of mean relative error of +/- 15% indicated by black dashed line. The mean relative error estimates are based on AU/mL values. (B) From the same data the within-lab geometric coefficient of variation (GCV) was calculated and plotted for each calibrator. Dashed black lines mark the acceptability criteria of a within-lab GCV of 15% or lower. Both the mean relative error estimates and the %GCV is calculated from AU/mL values.

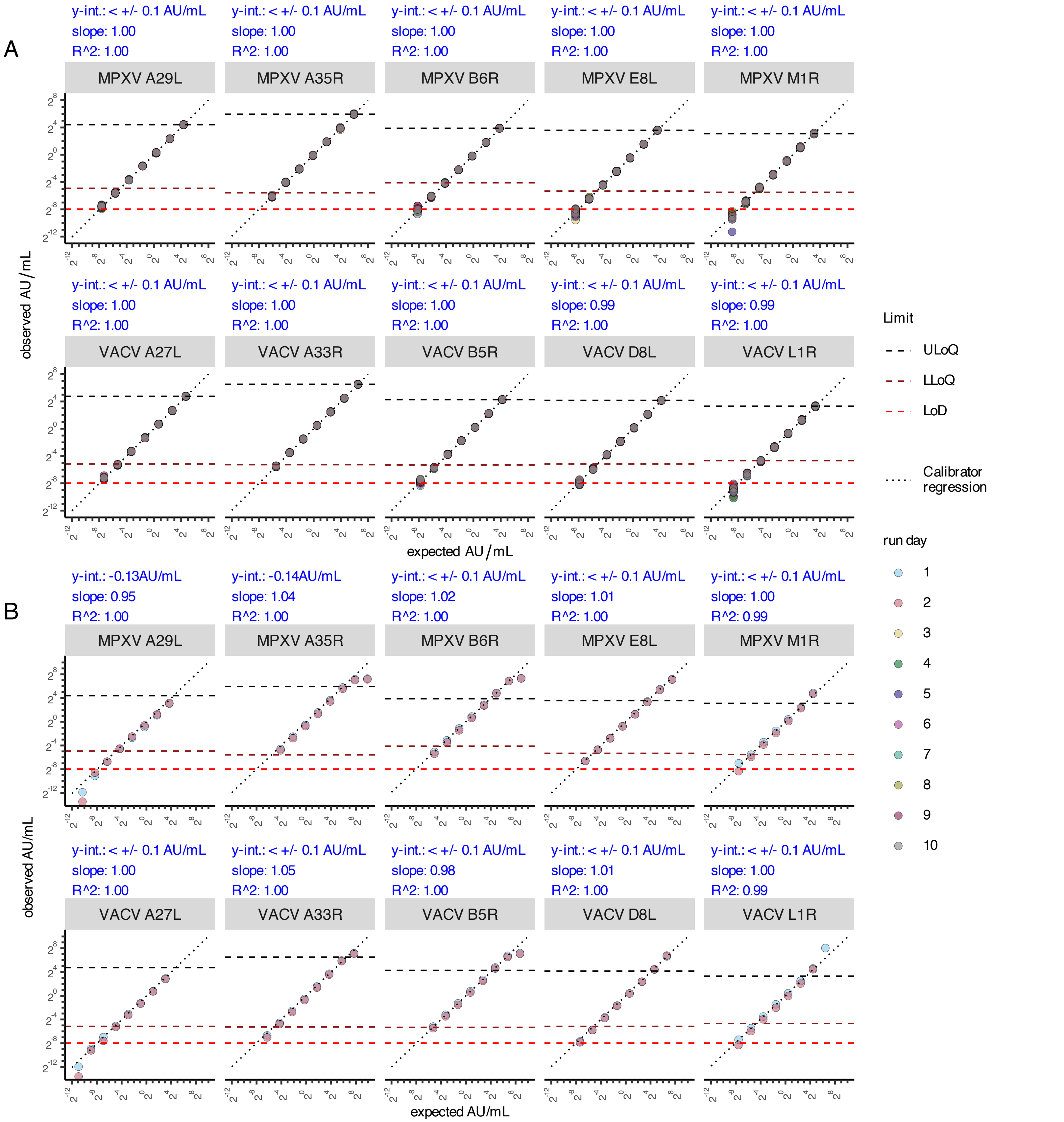

Figure S4: Antibody levels in the quantitative range of the MSD Orthopoxvirus assay are strongly linear. Linear regression was performed against observed (y-axis) and expected (x-axis) antibody levels (AU/mL) from testing (A) assay calibrators over ten days or (B) serially diluted serum specimen from MPXV-infected individual over two days (M32). Only results within the lower and upper limits of quantification were considered for linear regression (see Table S2). The expected AU/mL values for the calibrator are based on values provided by the manufacturer and the expected AU/mL values for the M32 specimen were based on testing of the M32 specimen in the MSD Orthopoxvirus assay at a dilution of 1/5000. The y-intercept, slope and R2 value determined from each linear regression is summarized in blue text above each plot. The dotted line represents the best fit line based on the linear regression of the calibrators only. The LoD, LLoQ, and ULoQ for each antigen are indicated in red, dark red, and black dashed lines, respectively.

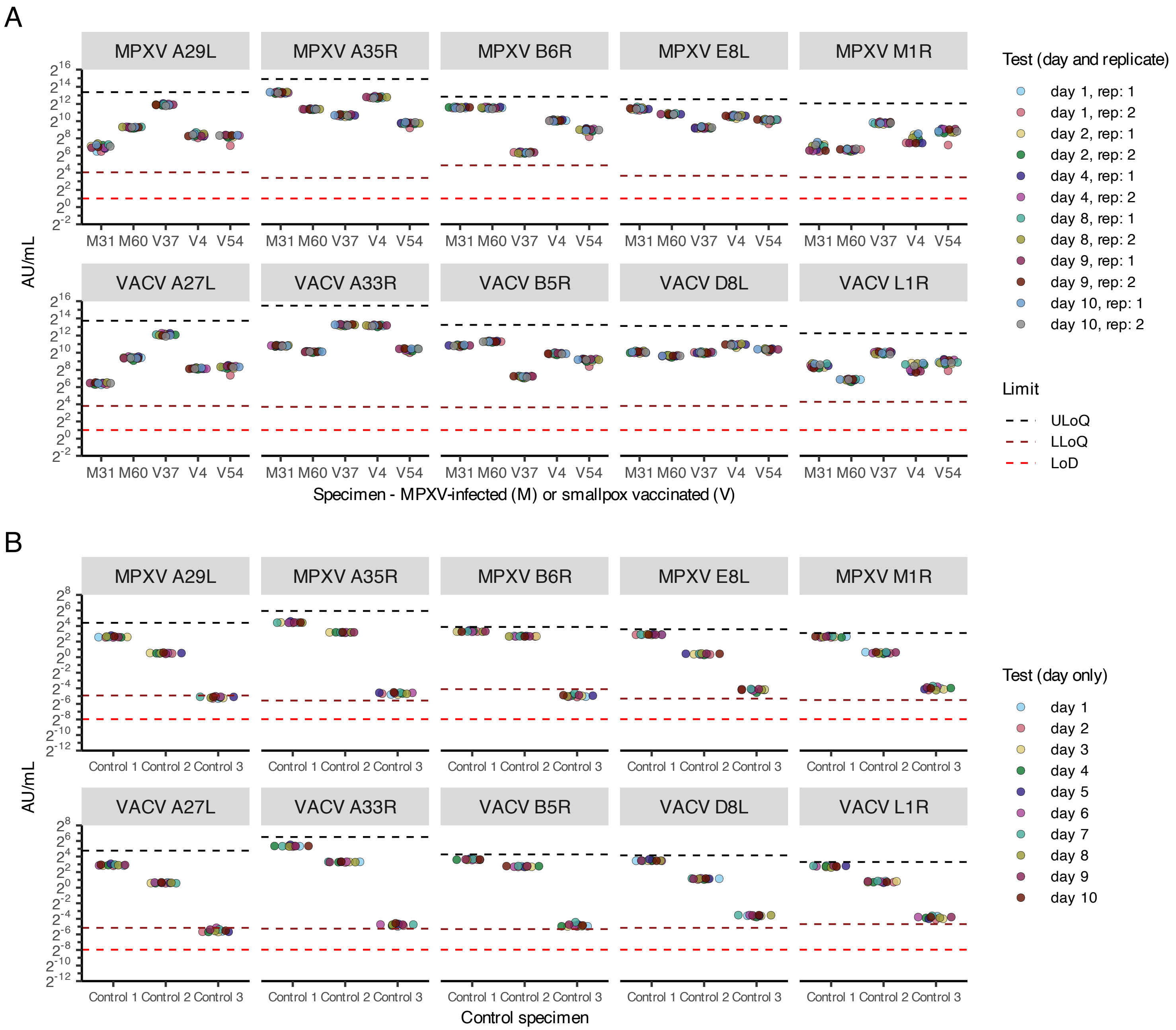

Figure S5: Antibody level (AU/mL) results for estimation of assay imprecision from either human serum specimens or serology controls provided by the manufacturer span the quantitative range of the assay. (A) Two serum specimens from MPXV-infected subjects (M) and three serum specimens from vaccinated subjects (V), which represent a range of antibody levels in the assay were selected for assessing assay imprecision. Specimens were tested over six days in duplicate, with reach replicate tested with two technical replicates (i.e. two wells per replicate) for a total of twelve average results per specimen. (B) Assay controls were tested once per run and the ten results shown are the average response from two wells. The ULoQ (black dashed line), LLoQ (brown dashed line), and LoD (red dashed line) established based on calibrator performance are shown. The LoD, LLoQ, and ULoQ are higher in (A) than (B) since human serum specimens were tested at a 500-fold dilution, whereas the serology controls were tested neat.

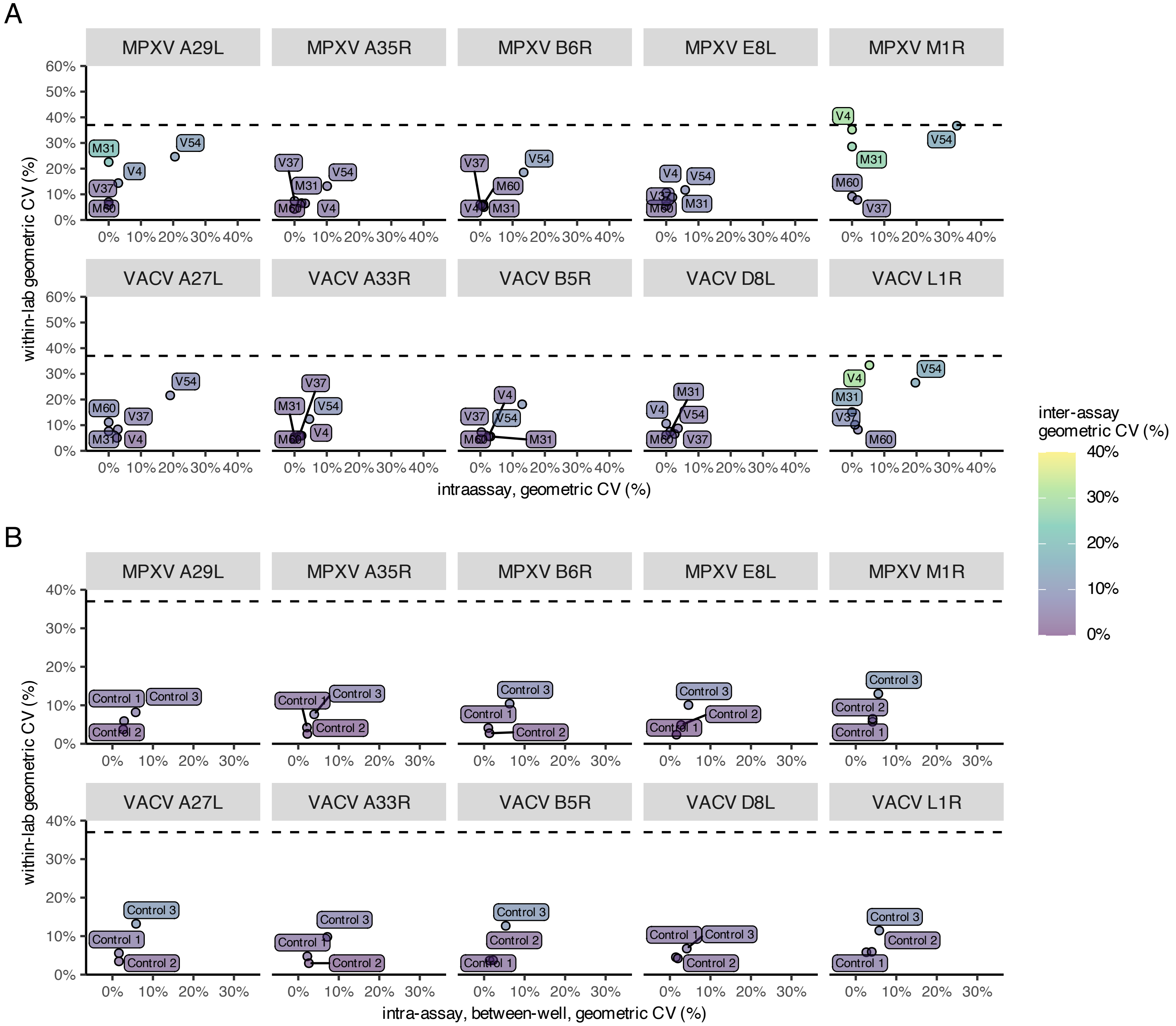

Figure S6: Imprecision of human serum specimens and serology controls meet acceptance criteria of within-lab geometric coefficient of variation (CV) of < 37%. Based on the testing shown in Figure S5, variance decomposition using ANOVA was performed to determine within-lab (y-axis), inter-assay (coloring of labels and points), and intra-assay imprecision (x-axis). (A) Are the imprecision results from testing human serum specimens shown in Figure S5A. Specimens IDs include whether there are from individuals MPXV-infected (M) or smallpox vaccinated (V). For specimen V4, a day 1 VACV L1R result and a day 10 MPXV M1R result, both with between-well geometric CV of greater than 37%, were removed for this analysis. (B) Are the imprecision results from testing the serology controls shown in Figure S5B. Since the serology controls were only tested once, with two technical replicates, the intraassay imprecision is between-well.

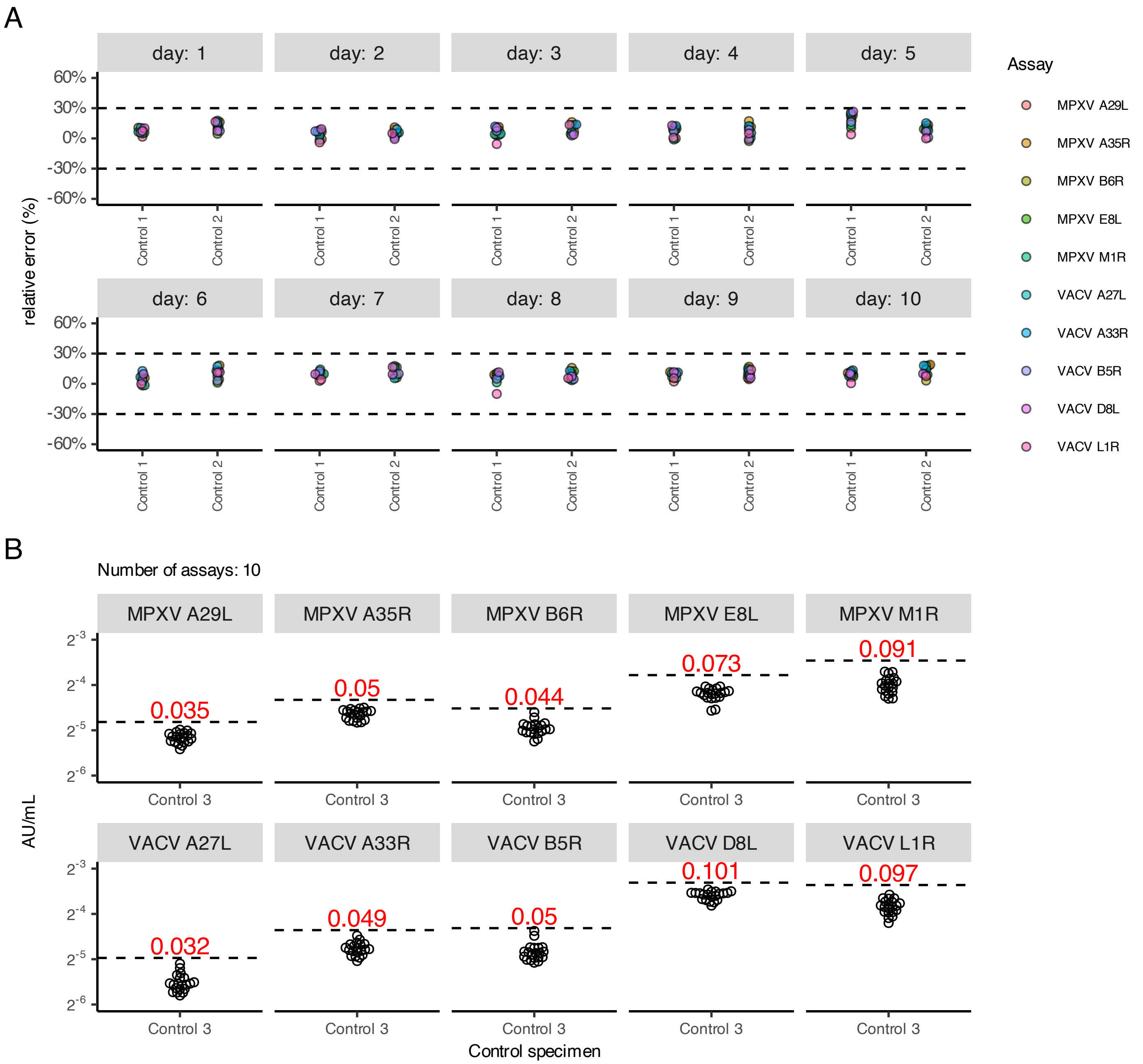

Figure S7: Based on manufacturer supplied serology controls the MSD Orthopoxvirus assay is accurate given that day-to-day relative error for Control 1 and 2 is less than +/- 30% and control 3 limit antibody levels are all less than 0.2 AU/mL. Serology controls (1,2 and 3) provided by the manufacturer were tested with two technical replicates over ten days. (A) Shown are the results for serology controls 1 and 2. The dashed black lines mark the acceptance criteria of a relative error of +/- 30%. (B) Shown are the results for serology control 3. The dashed black line is set to the control 3 geometric mean plus three times the control 3 geometric standard deviation. The acceptance criterion is meet since the Control 3 limit is less than 0.2 AU/mL for each antigen.

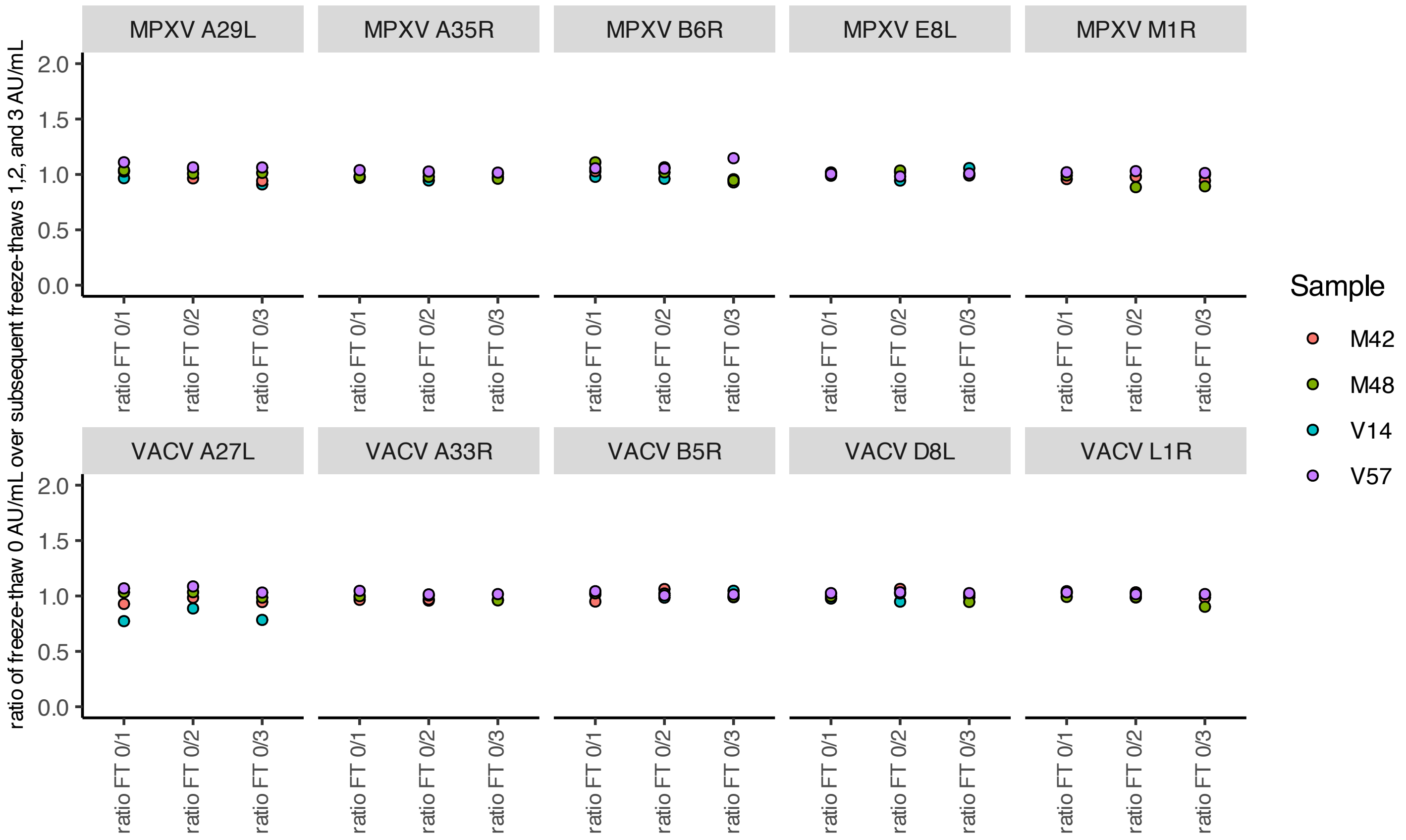

Figure S8: The assay is robust against freeze-thaw cycles of serum specimens. Plotted is the AU/mL ratio of the results before freeze-thaw (FT 0) over results from freeze-thaw 1 (ratio FT 0/1), from freeze-thaw 2 (ratio FT 0/2), or from freeze-thaw 3 (ratio FT 0/2). Shown are results from testing four specimens and specimens IDs include whether there are from individuals MPXV-infected (M) or smallpox vaccinated (V).

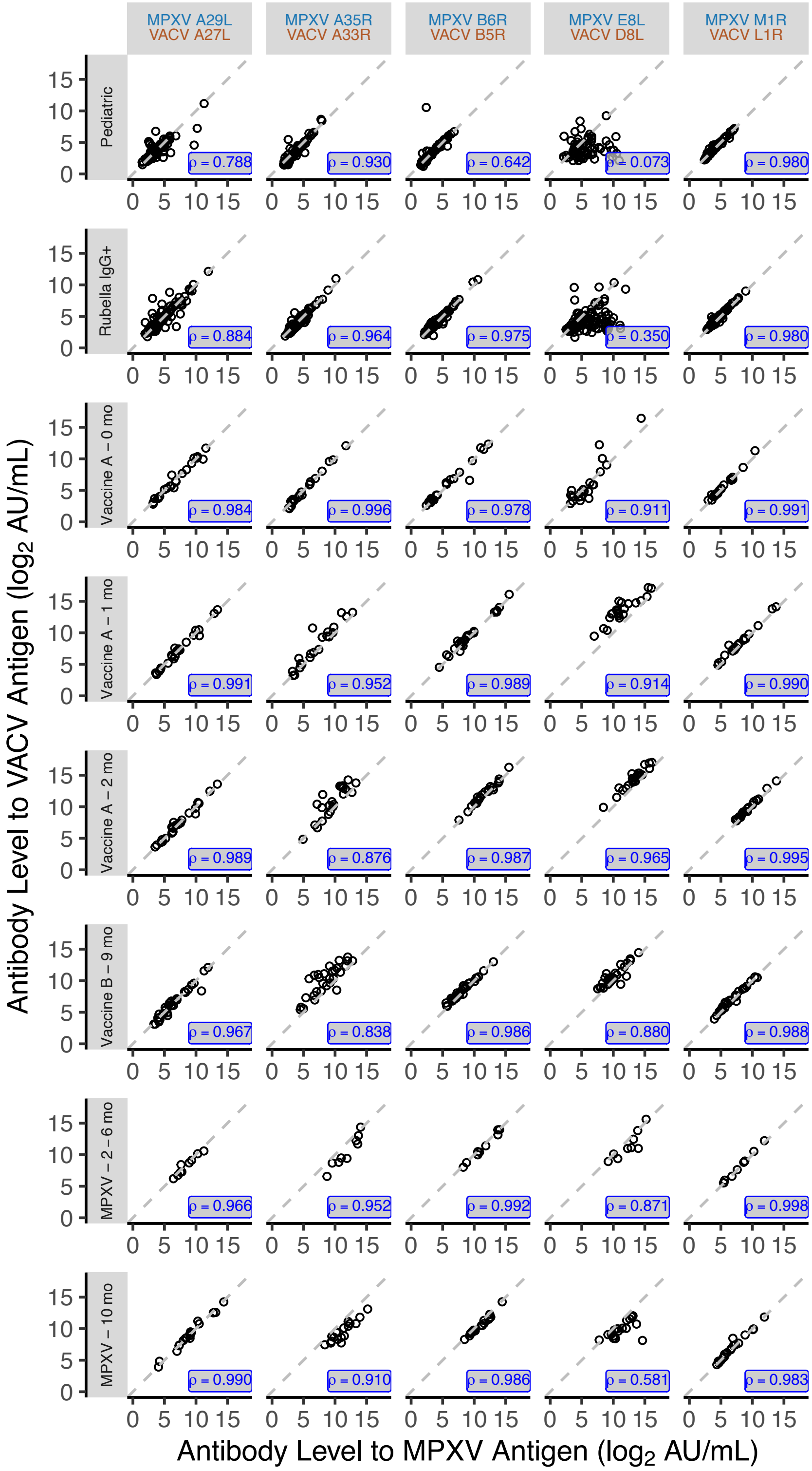

Figure S9: Antibody titer results against antigen orthologs are highly correlated. Shown are pairwise plots of the log2 transformed AU/mL results between antigen ortholog pairs within in each cohort. Inset value in blue is the Pearson's correlation coefficient, also shown in Figure 1C. The P61 MPXV B6R/VACV B5R results were omitted from the plot, since the VACV B5R antibody level was undetected.

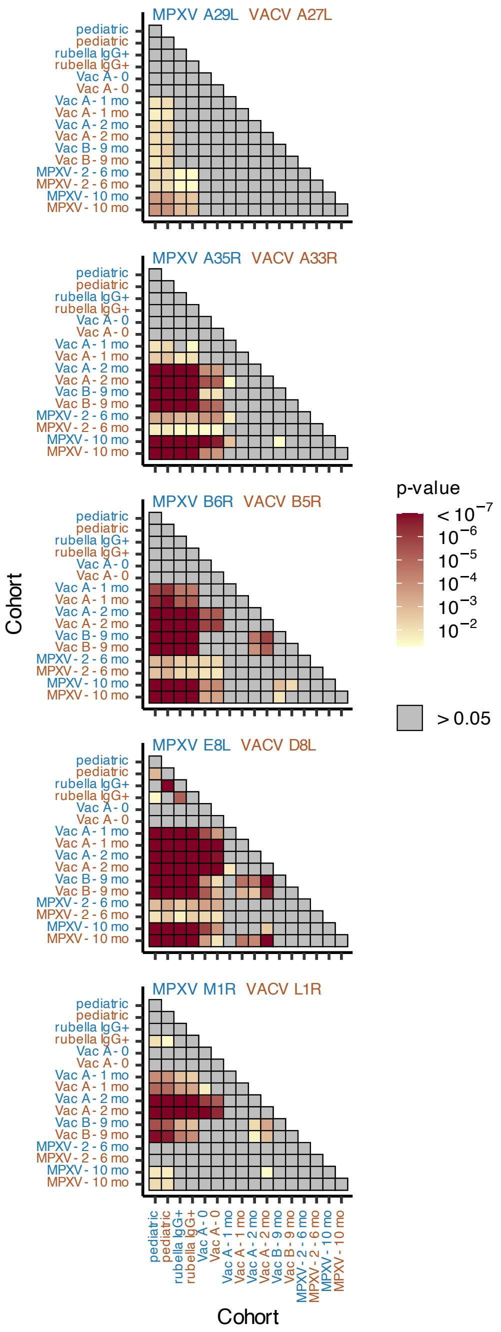

Figure S10. Significant differences in anti-orthopoxvirus antibody levels among MPXV-infected, vaccinated, and negative control cohorts. For each MPXV and VACV antigen pair (indicated along the top of each matrix), log-transformed antibody levels (AU/mL) were compared between cohorts using two-sided t-tests. Cohort names are color-coded: blue for MPXV antigen results and brown for VACV antigen results. The p-values are displayed in a lower-triangular matrix, where each tile represents a pairwise comparison as indicated by the intersecting column and row names. Tile shading intensity is inversely proportional to the p-value (i.e. darker red corresponds to a smaller p-value); gray tiles indicate p-value > 0.05. All p-values are Bonferroni-adjusted for multiple comparisons.

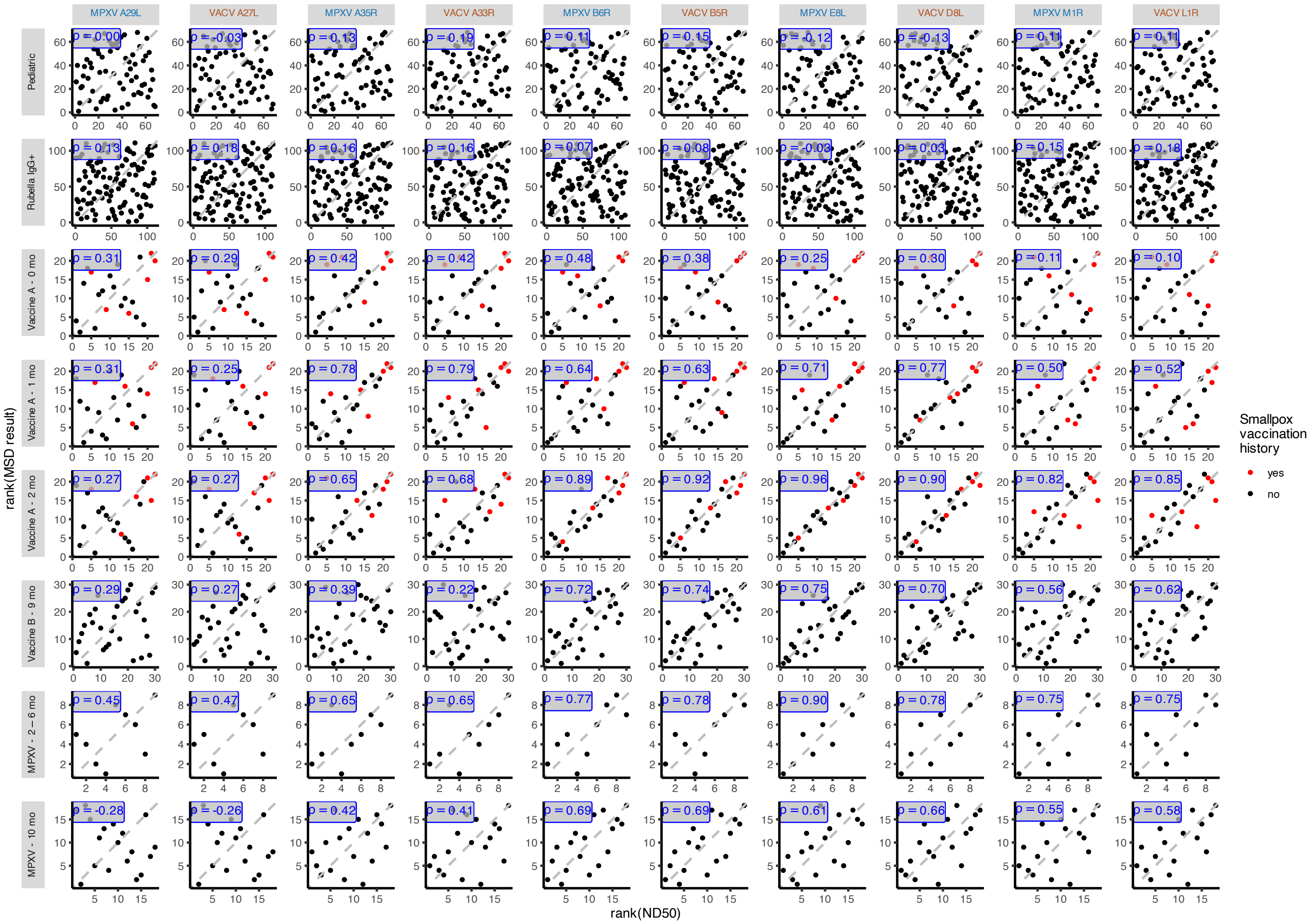

Figure S11: The anti-orthopoxvirus antibody titer results and MVA neutralization results are generally correlated in sera from individuals know to be MPXV infected or vaccinated. Shown are pairwise plots of the rank ordered MSD AU/mL results (MSD Response) verses rank ordered MVA neutralization assay ND50 results. Inset value in blue is the Spearman's correlation coefficient, also shown in Figure 4B. Results are filled with red if specimen is from an individual with known smallpox vaccination history.

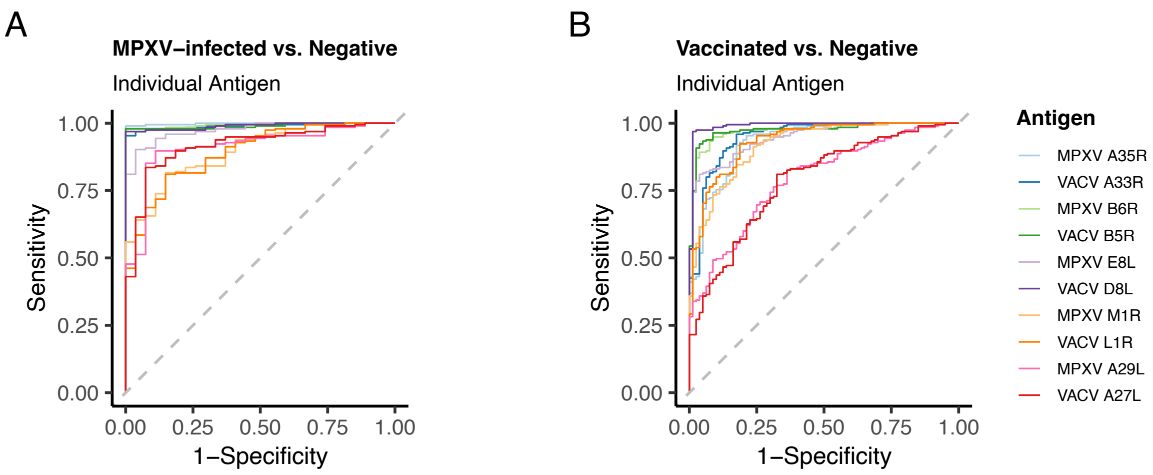

Figure S12: Receiver operator characteristic (ROC) curves separately comparing each exposed group (MPXV-infected or vaccinated) to negative controls. MPXV-infected refer to all sera from known MPXV infected individuals, Vaccinated refers to all sera from known vaccinated individuals, and Negative refer to all sera from the rubella and pediatric negative cohorts combined. Baseline sera collected prior to vaccination (Vaccine A – 0 month) were included in the Vaccinated group if they had a history of smallpox vaccination, otherwise they were categorized as negative (6 out of 22 individuals had previous history of vaccination). (A) ROC curves for all individual antigens generated by comparing all known MPXV-infected (n = 27) to all negatives (n = 195). (B) ROC curves for all individual antigens generated by comparing all known Vaccinated (n = 80) to all to all negatives (n = 195). Summary statistics from this analysis can be found Tables S6-S7.

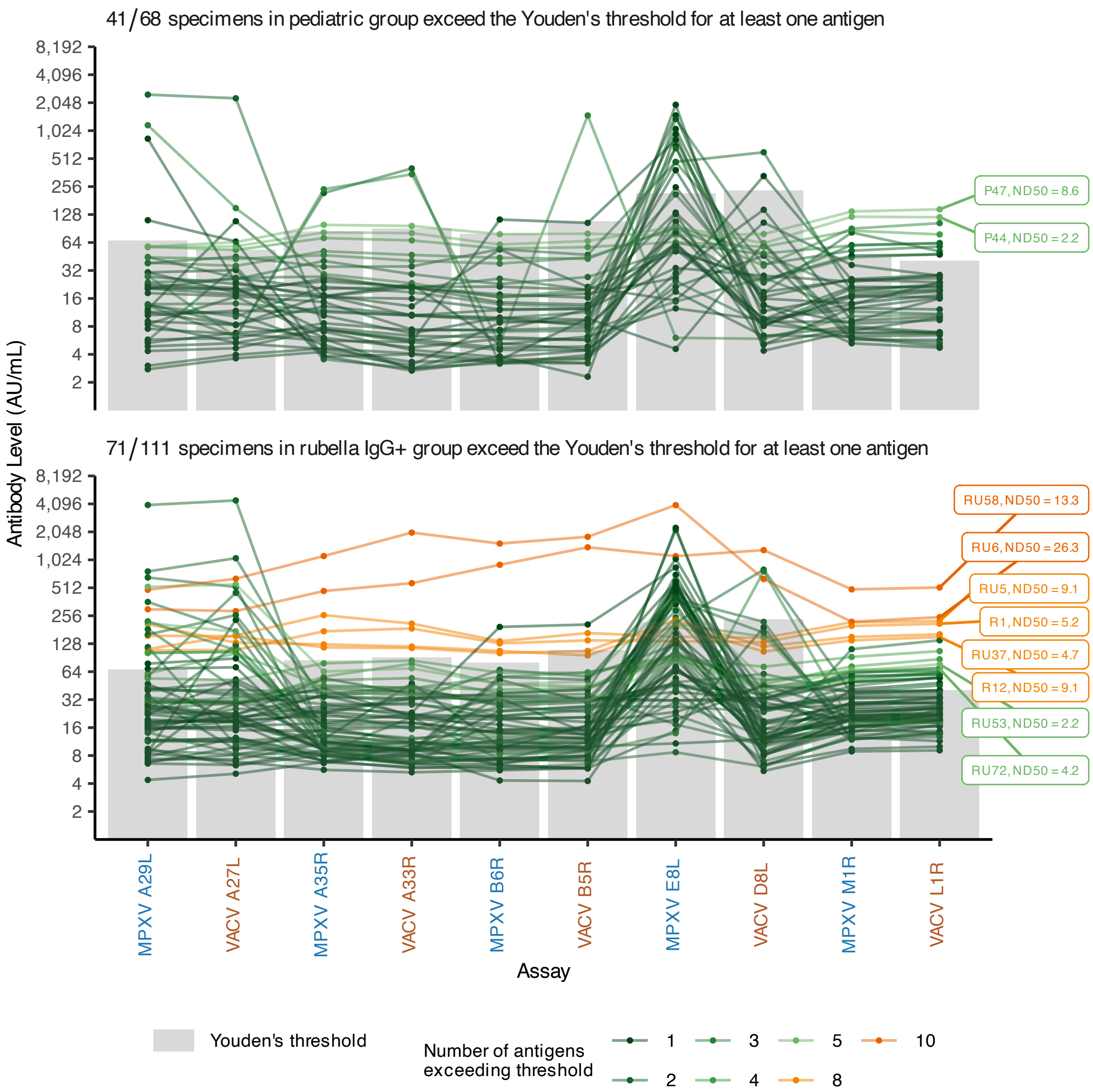

Figure S13: Some specimens in the negative cohorts (pediatric and rubella IgG+) exceed Youden's threshold (based on distinguishing exposed and unexposed groups) for one or more antigens. For each negative cohort (upper, pediatric; lower, rubella IgG+) the antibody level (AU/mL) is plotted for specimens that exceed Youden's threshold based on distinguish exposed vs. unexposed groups for at least one antigen (see Table S8). Specimens that exceeded the threshold for five or more antigens are labeled with the specimen identifier and MVA-neutralization ND50.
