# Supplementary material for: Differentiating mpox infection and vaccination using a validated multiplex orthopoxvirus IgG serology assay
Source: J Clin Microbiol. 2025 Dec 29;64(2):e01548-25. doi: 10.1128/jcm.01548-25 (PMC12892956; doi:10.1128/jcm.01548-25)
Supplement: Supplemental text — Supplemental material and methods. [file jcm.01548-25-s0002.docx]

**Supplementary Material**

**Validation**

**Analytical sensitivity**

The manufacturer does not specify a fixed limit of detection (LoD) for the VPLEX Orthopoxvirus assay, and instead the LoD is calculated for each run of the assay. The default method for calculating the LoD in the MSD Discovery Workbench 4.0 software utilizes the CAL-08 Signal results as follows:

LoD = mean_control_ x 2.5 x SD_control_; control is the specimen diluent alone (CAL-08)

Once the LoD is calculated, in terms of Signal, it is converted to AU/mL utilizing the calibrator curve. To establish a fixed LoD, calibrators were run in duplicate over ten days (Figure S1A for results in Signal and S1B for results in AU/mL). Comparison of Signal results for each of the calibrators over time were distinct across all calibrators for antigens MPXV A29L, VACV A27L, MPXV A35R, VACV A33R, and VACV D8L. In contrast, antigens MPXV B6R, VACV B5R, MPXV E8L, MPXV M1R, and VACV L1R there was overlap in Signal between calibrators over time, especially the lower concentration calibrators and the blank (CAL-05 thru CAL-07 and CAL-08). Standardization to AU/mL largely resolves this overlap since it normalizes Signal differences between runs except for CAL-07 and CAL-08 for some antigens (MPXV M1R and VACV L1R). The LoD was determined for each antigen using the MSD Discovery Workbench software over the ten runs and the geometric mean of the LoD for each antigen ranged from 0.0031 – 0.0038 AU/mL. Thus, we set the LoD for each antigen at 0.004 AU/mL (Table S2).

For quality control an upper limit was set for CAL-08 of a Signal of 300 based on the range of values observed during our validation runs (Figure S2 – validation runs annotated with light blue bar). Notably, during validation, four out of ten runs exhibited higher CAL-08 Signal relative to the other runs for the MPXV M1R and VACV L1R antigens. For these antigens, the percent relative error (%RE) of the control materials provided by the manufacture were within +/- 30% for control 1 and 2 and < 0.2 AU/mL for control 3 and the geometric standard deviation of the control materials were all within 1.42. Thus, despite the slightly elevated CAL-08 Signal the assays were considered valid. During subsequent testing, several assays were flagged for having CAL-08 Signal exceeding a Signal of 300, especially for the MPXV M1R and VACV L1R antigens (Figure S2 between Oct 2024 and Jan 2025). Again, the manufactures controls performed as expected based on criteria described above. Plate images and data were provided to the manufacturer, and the manufacturer could not identify any artifacts (e.g. well drying) or manufacturing correlates that could explain the elevated CAL-08 Signal. All assays were performed by two operators using the same lot number of materials. To ensure sufficient dynamic range in Signal, the criteria was changed to ensure at least a 200-fold difference between CAL-01 and CAL-08 signals, rather than utilizing an absolute value for CAL-08 Signal. Based on this criterion, out of 75 assays, four assays failed for the M1R antigen and one assay failed for both M1R and L1R antigen (Figure S2, red-filled points).

**Verification of Calibration model**

The calibration model was verified by testing of the calibrators over ten days. The day-to-day percent relative error was within +/- 30% for all calibrators, except CAL-07 which was inconsistent from day-to-day for some antigens (data not shown). The average percent relative error observed over ten days of testing was within +/- 15% (Figure S3A). Finally, within-lab imprecision was within 15% geometric coefficient of variation (GCV) for all calibrators, except CAL-07 which was greater than GCV of 15% for a subset of the antigens (Figure S3B). Lastly, the four-parameter logistic regression (4PL) fits for each antigen’s calibration curve had R^2^ values > 0.9 (Table S3). Taken together, these data verify the validity of the calibration model and that CAL-07.

Based on the imprecision of the calibrators shown here the lower limit of quantification (LLoQ), was less than the LLoQ reported by the manufacturer (Table S2). The more conservative manufacturers estimates were selected as the preliminary LLoQ (see Table S2, LLoQ final column). The ULoQ was set to the mean back-calculated AU/mL value of the highest calibrator (CAL-01) (see Table S2, ULoQ final avg. calculated AU/mL of CAL-01).

**Linearity**

To assess linearity the results within the limits of quantification (LoQ) from testing of the assay calibrators over ten days were subjected to linear regression analysis (Figure S4A). For all antigens, the calibrators demonstrated strong linearity with R² values exceeding 0.9. Furthermore, for all antigens, the slopes of the linear regression were approximately one, and the y-intercepts were below 0.2 AU/mL, indicating the absence of systemic or proportional bias.

We also assessed linearity with strongly positive serum specimen (M32) that was previously tested in the V-PLEX Orthopoxvirus assay. The specimen was initially diluted 50-fold followed by seven 4-fold dilutions. Each dilution was measured in the assay and the back-calculated AU/mL values, not corrected for dilution, were subjected to linear regression (Figure S4B). Like the calibrators, the M32 dilution series was strongly linear with R² values exceeding 0.9. Furthermore, for all antigens, the slopes of the linear regression were approximately one, and the y-intercepts were below 0.2 AU/mL, indicating the absence of systemic or proportional bias. Lastly, the M32 dilution series results were highly parallel to the calibrator linear regression (Figure S4B, dotted line corresponds to the calibrator linear regression best fit), supporting the use of the calibrators and selected diluent in estimating AU/mL levels from serum specimens. Similar results were obtained with Reference Vaccinia Immune Globulin (VIGIG) pooled human serum from CBER/FDA lot 1 (data not shown).

**Precision**

Assay imprecision was estimated from two data sets. First, two human serum specimens from MPXV-infected subjects and three human serum specimens from Jynneos-vaccinated subjects were selected. Each specimen was tested in duplicate, with each replicate tested utilizing two wells, across six separate days (Figure S5A). These specimens spanned a broad range of antibody concentrations within the assay’s quantifiable limits, as determined by calibrator performance. Second, results from testing the manufacturer supplied serology controls (1,2, and 3), with two technical replicates (two wells), over ten days were also analyzed (Figure S5B). Collectively, the three controls span the assay’s quantitative range. Control 3 fell just below the lower limit of quantification (LLoQ) for MPXV A29L, VACV A27L, and MPXV B6R antigens, which is expected since control 3 is presumed negative. Variance decomposition was performed using ANOVA to estimate intra-assay, inter-assay, and within-laboratory components. Results with an inter-well geometric coefficient of variation (GCV) > 37% were excluded, as this level of variability between technical replicates may indicate an unreliable measurement. Only human serum specimen V4 was affected, with one VACV L1R result (day 1) and one MPXV M1R result (day 10) removed. All human serum specimens demonstrated within-laboratory imprecision with GCV <37% (Figure S6A). For the serology controls, despite some control 3 results falling below the LLoQ, all three controls exhibited within-laboratory imprecision with GCV < 37% (Figure S6B). Notably, the serology controls showed lower and more consistent imprecision compared to the selected specimens described above, so they don’t appear to entirely reflect real-world variability.

**Accuracy**

Accuracy was evaluated by comparing measured results from the manufacturer-supplied serology controls (Control 1, Control 2, and Control 3) to their manufacturer assigned AU/mL values. Control 1 and Control 2 are expected to fall within the high and mid quantitative ranges of each assay (Figure S5B). Each control was tested in duplicate wells across ten independent runs. For both Control 1 and Control 2, the relative error compared to the manufacturer-assigned AU/mL values was within ±30% for all assays (Figure S7A). Control 3 serves as a presumptive negative control, with assigned AU/mL values of < 0.1 AU/mL for VACV A27L, MPXV A29L, VACV A33R, MPXV A35R, VACV B5R, MPXV B6R, VACV D8L, and MPXV E8L, and < 0.2 AU/mL for VACV L1R and MPXV M1R. Control 3 was tested alongside the other controls in duplicate wells across ten runs (Figure S5B). The geometric mean response and geometric standard deviation (GSD) were calculated, and an empirical limit for Control 3 was determined by adding three times the GSD to the geometric mean (limits shown in red in Figure S7B). All empirically determined limits for Control 3 were ≤ 0.1 AU/mL. Together, these results demonstrate the accuracy of the assay relative to the manufacturer-supplied serology controls (Controls 1–3) since the percent relative error of control 1 and 2 are < ± 30% and Control 3 AU/mL values were ≤ 0.1 AU/mL.

**Analytical Measurement Range**

The lower limit of quantification (LLoQ) was defined as the minimum concentration above the limit of detection (LoD) that meets precision criteria and falls within the assay’s linear range. The upper limit of quantification (ULoQ) was defined as the maximum concentration that meets precision criteria and remains within the linear range. Together, the LLoQ and ULoQ establish the analytical measurement range (AMR) of the assay. All calibrator-derived results within the LLoQ and ULoQ met acceptance criteria for both linearity (Figure S4) and imprecision (Figures S5 and S6). Therefore, the LLoQ and ULoQ values presented in Table S2 define the assay’s validated AMR.

**Robustness**

Since clinical specimens are likely to undergo several freeze thaws (FT) between collection and testing, the effect of FT on the assay results was evaluated. Four specimens were selected, two MPXV-infected (M42, M48) and two smallpox vaccinated (V14, V57). Each specimen was thawed and four aliquots prepared and frozen back down. One tube was designated as freeze-thaw (FT) 0 and not thawed again till testing, one tube was designated as FT 1 and was thawed once and frozen down again prior to testing, one tube was designated as FT 2 and was thawed and frozen twice prior to testing, one tube was designated as FT 3 and was thawed and frozen three times prior to testing. The ratio of antibody level results between FT 0 and each of the different freeze-thaw specimens (1-3), was calculated and all ratios were within 0.5 -1.5-fold, so no significant freeze-thaw effects were detected (Figure S8).

**Supplementary Methods**

*Production of MVA Virus*

Vaccinia virus Modified Vaccinia Ankara strain (MVA) (BEI, NR-727) was propagated in BHK-21 cells (ATCC, CCL-10) at a multiplicity of infection (MOI) of 0.7. Cultures were harvested 72 hours post-infection, when approximately 80% of cells exhibited cytopathic effects, by scraping cells into the culture supernatant and transfer of the entire cell suspension to a 15 mL conical on ice. The cell suspension was centrifuged at 1,200 × g for 10 minutes at 4 °C to separate cell debris, and the resulting supernatant (S0) was transferred to a 15 mL conical tube and kept on ice. The cell pellet was resuspended in 1 mL of S0 and subjected to a freeze-thaw cycle by immersion in liquid nitrogen for 1–2 minutes, followed by rapid thawing at 37 °C. The resuspended freeze-thawed pellet was briefly vortexed and centrifuged again at 1,200 × g for 10 minutes at 4 °C. The supernatant from the freeze-thaw (FTS) was collected into a separate 15 mL conical tube on ice. This freeze-thaw procedure was repeated two additional times. Finally, all collected FTS supernatants were pooled and combined with the remaining S0.

*Vaccinia Virus Focus Reduction Neutralization Test Qualification*

The MVA neutralization assay was qualified. Briefly, precision was estimated and found acceptable (geometric standard deviation of the 50% neutralizing titer (ND50) < 1.42) by repeat testing specimens M33, V47, M48, M50, M8 in duplicate over three days (data not shown). The CBER Vaccinia IgG Reference Standard, Lot 1, yielded an average ND50 of 1,551, equivalent to 41.3 µg/mL, consistent with previously reported values of 24–49 µg/mL (1). The lower limit of quantification (LLoQ) of the MVA neutralization was defined by the lowest dilution tested.

*Meso Scale Discovery V-PLEX Multispot Orthopoxvirus Serology Assay Validation*

A detailed description of the MSD assay validation is provided in the Supplementary material. For precision testing, serum specimens V4, M31, V37, V54, and M60 (selected from the Vaccine B and MPXV–10-month cohorts) were thawed and single-use aliquots prepared for analysis. In addition to the calibrators, two additional specimens were used to assess linearity, M32 (from MPXV-10-month cohort) and Reference Vaccinia Immune Globulin (VIGIG), lot 1 from CBER/FDA (VIGIG). M32 was initially diluted 5-fold and VIGIG diluted 50-fold to bring the materials near the linear range of the assay. For testing, pre-diluted linearity specimens were diluted and tested following the same procedure as the calibrators. Table S1 summarizes the limit of detection (LoD), lower limit of quantification (LLoQ), and upper limit of quantification (ULoQ) for each antigen.

**Supplemental References**

1. Taub DD, Ershler WB, Janowski M, Artz A, Key ML, McKelvey J, Muller D, Moss B, Ferrucci L, Duffey PL, Longo DL. 2008. Immunity from Smallpox Vaccine Persists for Decades: A Longitudinal Study. Am J Med 121:1058–1064.
